# Engineering *Clostridium Tyrobutyricum* for High Butanol Production through Induction Expression of Exogenous NADPH-Dependent HBD

**DOI:** 10.1021/acssynbio.5c00792

**Published:** 2026-03-11

**Authors:** Qingke Wang, Geng Wang, Jialei Hu, Jun Feng, Ziqi Emily Lin, Shang-Tian Yang

**Affiliations:** William G. Lowrie Department of Chemical and Biomolecular Engineering, 2647 The Ohio State University, Columbus, Ohio 43210, United States

**Keywords:** 3-hydroxybutyryl-CoA dehydrogenase, butanol, carbon flux, Clostridium tyrobutyricum, inducible gene expression, metabolic engineering

## Abstract

*Clostridium tyrobutyricum* Δ*cat*1::*adh*E2 is a promising cell factory for butanol production because of its robustness, high butanol tolerance, and minimal butyrate production. However, excessive acetate and ethanol production remains a major bottleneck limiting its butanol yield. Coexpressing an exogenous *hbd*(*Ck*) encoding the NADPH-dependent 3-hydroxybutyryl-CoA dehydrogenase (HBD) from *Clostridium kluyveri* with *adh*E2 could increase the C4 carbon flux, resulting in increased butanol and decreased acetate and ethanol production. However, constitutively overexpressing *hbd*(*Ck*) in Δ*cat*1::*adh*E2 shows little improvement in butanol yield, productivity, and selectivity, which might be caused by redox imbalance and growth inhibition. To alleviate this problem, *C. tyrobutyricum* MΔ*cat*1::*adh*E2-P*bgal*-*hbd*(*Ck*) with a dynamic expression of *hbd*(*Ck*) controlled by an inducible promoter was developed. In serum bottle fermentation at 37 °C, when the *hbd*(*Ck*) expression was induced at 12 h or in the early exponential phase, butanol production increased ∼20% in yield (from 0.22 to 0.27 g/g glucose), 87.5% in productivity (from 0.16 to 0.30 g/L·h), and 52% in selectivity (from 0.46 to 0.70 g/g total products) compared to the control strain without expressing any *hbd*(*Ck*), whereas *hbd*(*Ck*) expression induced at 0 or 24 h in MΔ*cat*1::*adh*E2-P*bgal*-*hbd*(*Ck*) or constitutively in MΔ*cat*1::*adh*E2-P*cat*1-*hbd*(*Ck*) showed significantly lower butanol yield and productivity. At 25 °C, MΔ*cat*1::*adh*E2-P*bgal*-*hbd*(*Ck*) with 12 h induction produced the highest butanol titer of 23 g/L with 0.32 g/g yield, 0.16 g/L·h productivity, and 0.83 g/g product selectivity due to much reduced acetate formation. Subsequent scale-up to a stirred-tank bioreactor at 37 °C increased productivity to 0.39 g/L·h while also achieving high butanol titer (21.8 g/L), yield (0.30 g/g), and selectivity (0.67 g/g). The optimized induction timing resulted in a balanced NAD­(P)H pool, effectively channeling substrates toward butanol biosynthesis. It was concluded that the timing for *hbd*(*Ck*) expression was critical as it affected glucose catabolism, cell growth, redox balance, and carbon flux distribution. These findings underscore the potential of dynamic metabolic regulation to overcome bottlenecks in biobutanol production, providing a scalable and economically viable bioprocess for industrial application.

## Graphical abstract

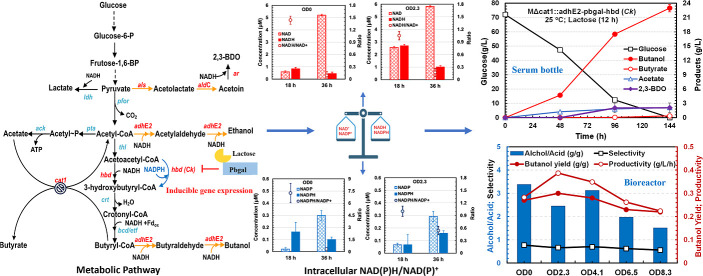

## 1 Introduction

Biobutanol is an important industrial solvent and a potential drop-in biofuel for automobiles.^1,2^ Traditional solventogenic clostridia naturally produce butanol via the well-known ABE fermentation pathway.^3–5^ Despite their natural ability to produce solvents, solventogenic clostridia give relatively low butanol yield, productivity, and titer due to the coproduction of acetone and ethanol and poor butanol tolerance, which restrict their industrial applications.^6–9^ The two-phase ABE fermentation by solventogenic clostridia is also difficult to control for long-term operation in a (semi)­continuous process^10,11^ because of spontaneous sporulation and culture degeneration.^12–14^ More recently, *Clostridium tyrobutyricum* was engineered to overexpress *Clostridium acetobutylicum* *adh*E2 encoding the bifunctional aldehyde/alcohol dehydrogenase (AAD) for converting butyryl-CoA to butyraldehyde and then to butanol and has attracted considerable attention as a promising bacterial platform for butanol production due to its high butanol tolerance.^15–17^ While butyrate and acetate are two major byproducts limiting butanol yield in fermentation by the engineered *C. tyrobutyricum* overexpressing *adh*E2, further metabolic engineering and process optimization allowed it to produce butanol at high titer, rate (productivity), and yield (TRY) (15–20 g/L, ∼0.3 g/L·h, and ∼0.3 g/g, respectively) from glucose and biomass hydrolysate sugars.^18–21^ Furthermore, genome-engineered *C. tyrobutyricum* Δ*cat*1::*adh*E2, which was developed using CRISPR-Cas technology for *adh*E2 insertion on the genome with simultaneous knockout of *cat*1 encoding the butyryl-CoA/acetate CoA transferase responsible for the biosynthesis of butyric acid, produced butanol at a yield of 0.23 g/g.^22,23^ Although little butyrate was produced by Δ*cat*1::*adh*E2, the coproduction of acetate and ethanol in large amounts limited butanol yield to a level far below the theoretical 0.41 g/g glucose. To date, it remains a challenge to engineer *C. tyrobutyricum* with butanol as the primary or sole product without much coproduction of acetate and butyrate.

In solventogenic clostridia, HBD converts acetoacetyl-CoA to 3-hydroxybutyryl-CoA with NADH as the redox cofactor, which could limit the flux toward C4 metabolites and butanol biosynthesis. Overexpressing an exogenous *hbd*(*Ck*) encoding the NADPH-dependent HBD from *Clostridium kluyveri* was shown to increase butanol biosynthesis in *C. acetobutylicum* ^24^ and engineered *Clostridium cellulovorans* ^25^ and *C. tyrobutyricum* overexpressing *adh*E2.^26^ Compared to the strains expressing only *adh*E2, coexpressing *adh*E2 and *hbd*(*Ck*) in *C. tyrobutyricum* WT and Ack strains produced significantly more butanol due to increased C4 flux and reducing equivalents.^26^ However, overexpressing *hbd*(*Ck*) in Δ*cat*1:*adh*E2 did not show any significant effects on flux redistribution and butanol biosynthesis.^26^ The intracellular concentrations of NAD­(P)­H/NAD­(P)^+^ play a critical role in regulating bacterial growth and metabolism.^27^ Overexpressing NADPH-dependent HBD in *C. tyrobutyricum* Δ*cat*1::*adh*E2 might disrupt its NADH/NAD^+^ homeostasis, resulting in inefficient glycolysis and poor cell growth and metabolic activities. Dynamic metabolic regulation through inducible gene expression systems to control temporal expression of metabolic enzymes and optimize their activity to specific growth stages or fermentation phases may resolve such limitations and alleviate metabolic burden and redox imbalance, resulting in improved product titer, yield, and productivity.^28,29^

In this study, we investigated the use of an inducible promoter P*bgal* ^30^ for the dynamic expression of *hbd*(*Ck*) in *C. tyrobutyricum* MΔ*cat*1::*adh*E2. The lactose inducible promoter P*bgal* was chosen as it has a high dynamic range of 40-fold change in the gene expression level induced with lactose at ∼ 40 mM.^30^ Other inducible expression systems, including IPTG and tetracycline inducible promoters, have also been explored for various *Clostridium* spp., but their compatibility and efficacy with *C. tyrobutyricum* have not been verified. We aimed to optimize butanol production by controlling the expression of *hbd*(*Ck*) in *C. tyrobutyricum* MΔ*cat*1::*adh*E2-P*bgal*-*hbd*(*Ck*) at varying induction times (0, 12, and 24 h) during batch fermentation. Additionally, we evaluated the impacts of methyl viologen (MV) and low-temperature culture for their effects on maximizing metabolic flux toward butanol biosynthesis. Finally, we scaled up the batch fermentation with *C. tyrobutyricum* MΔ*cat*1::*adh*E2-P*bgal*-*hbd*(*Ck*) in a stirred-tank bioreactor with *hbd*(*Ck*) expression induced in the early exponential phase, which provided a balanced NAD­(P)H pool to effectively channel substrates toward butanol biosynthesis without impeding cell growth and achieved high butanol titer, productivity, and yield (21.8 g/L, 0.39 g/L·h, and 0.30 g/g, respectively) suitable for industrial biobutanol production. This study contributed to the development of next-generation recombinant clostridia strains for efficient, high-yield, and economically viable biobutanol production.

## 2 Results

### 2.1 Inducible Expression of hbd­(Ck) at Different times in Batch Fermentation

Previous studies demonstrated that overexpressing *hbd*(*Ck*) increased the carbon flux from acetyl-CoA to butyryl-CoA and butanol production in *C. tyrobutyricum* strains WT-*adh*E2, Ack-*adh*E2 and Δ*hyd*A-*adh*E2 but not in Δ*cat*1::*adh*E2.^26^ Compared to MΔ*cat*1::*adh*E2, MΔ*cat*1::*adh*E2-P*cat*1-*hbd*(*Ck*) overexpressing *hbd*(*Ck*) using the constitutive *cat*1 promoter showed slightly slower glucose consumption and butanol production with minimal effect on acetate production (Figure 1A,B). Constitutive expression of NADPH-dependent HBD likely perturbed the redox balance by redirecting NADPH for 3-hydroxybutyryl-CoA formation from biosynthesis/anabolism for cell growth.^26^ We thus hypothesized that overexpressing the NADPH-dependent HBD from the beginning of the batch culture might have limited NAD^+^ availability for glycolysis, depleted the NADPH pool needed for cell growth, and caused NADH/NADPH imbalance that resulted in decreased cell growth and butanol production in Δ*cat*1::*adh*E2-*hbd*(*Ck*). To address this issue, we constructed the strain MΔ*cat*1::*adh*E2-P*bgal*-*hbd*(*Ck*) with the lactose-inducible promoter P*bgal* ^30^ for controlling the expression of *hbd*(*Ck*) and investigated the effect of induction timing of *hbd*(*Ck*) expression on butanol production in batch fermentation. As can be seen in Figure 1C–E, *hbd*(*Ck*) induction timing showed significant effects on butanol and acetate production. Induction at 12 h gave the highest butanol production (12.6 g/L) with significantly reduced acetate production (5.1 g/L), while induction at 0 or 24 h showed negligible benefits in reducing acetate or increasing butanol production. Adding lactose at 0 h to induce *hbd*(*Ck*) expression immediately after inoculation led to a large amount of acetate produced (11.3 g/L) that surpassed butanol production (10.5 g/L) and resulted in low butanol yield (0.19 ± 0.01 g/g) and productivity (0.18 ± 0.02 g/L·h), which were comparable to or somewhat lower than those from the strains without *hbd*(*Ck*) expression or with constitutive *hbd*(*Ck*) expression (Figure 1F). On the other hand, induction at 24 h showed minimal effect on reducing acetate production and produced butanol at a comparable or slightly lower amount than those from Δ*cat*1::*adh*E2 and Δ*cat*1::*adh*E2-P*cat*1-*hbd*(*Ck*). In contrast, induction at 12 h significantly reduced acetate and increased butanol production, resulting in a significantly higher butanol yield (0.23 ± 0.01 g/g) and productivity (0.26 ± 0.01 g/L·h). The impact of the optimized induction time (12 h) for *hbd*(*Ck*) expression on flux distribution was also evident from the dramatic increase (∼100%) in the ratios of alcohol to acid [(butanol + ethanol)/(acetate + butyrate)] and C4/C2 [(butanol + butyrate)/(ethanol + acetate)], which increased to ∼2.4 g/g from ∼1.3 g/g (Figure 1F). Consequently, the product selectivity (butanol/all metabolic products) increased to >0.7 (vs < 0.5 for other conditions). The higher butanol yield, productivity, and selectivity for fermentation with lactose induction at 12 h implied that more NADH was used for butanol biosynthesis (Figure 1G), which was estimated from the product yields using the stoichiometric equations given in Table S2. It is noted that significant 2,3-BDO production (∼2 g/L) was observed in these batch fermentations. However, the fermentation with *hbd*(*Ck*) induction at 12 h produced significantly less amounts of 2,3-BDO, which was eventually converted to butanol by the end of the batch fermentation (Figure 1D). 2,3-BDO formation could function as an overflow/redox-balancing sink from pyruvate under certain redox and flux states. However, the exact mechanism is not clear and requires further studies.

**Figure 1:**
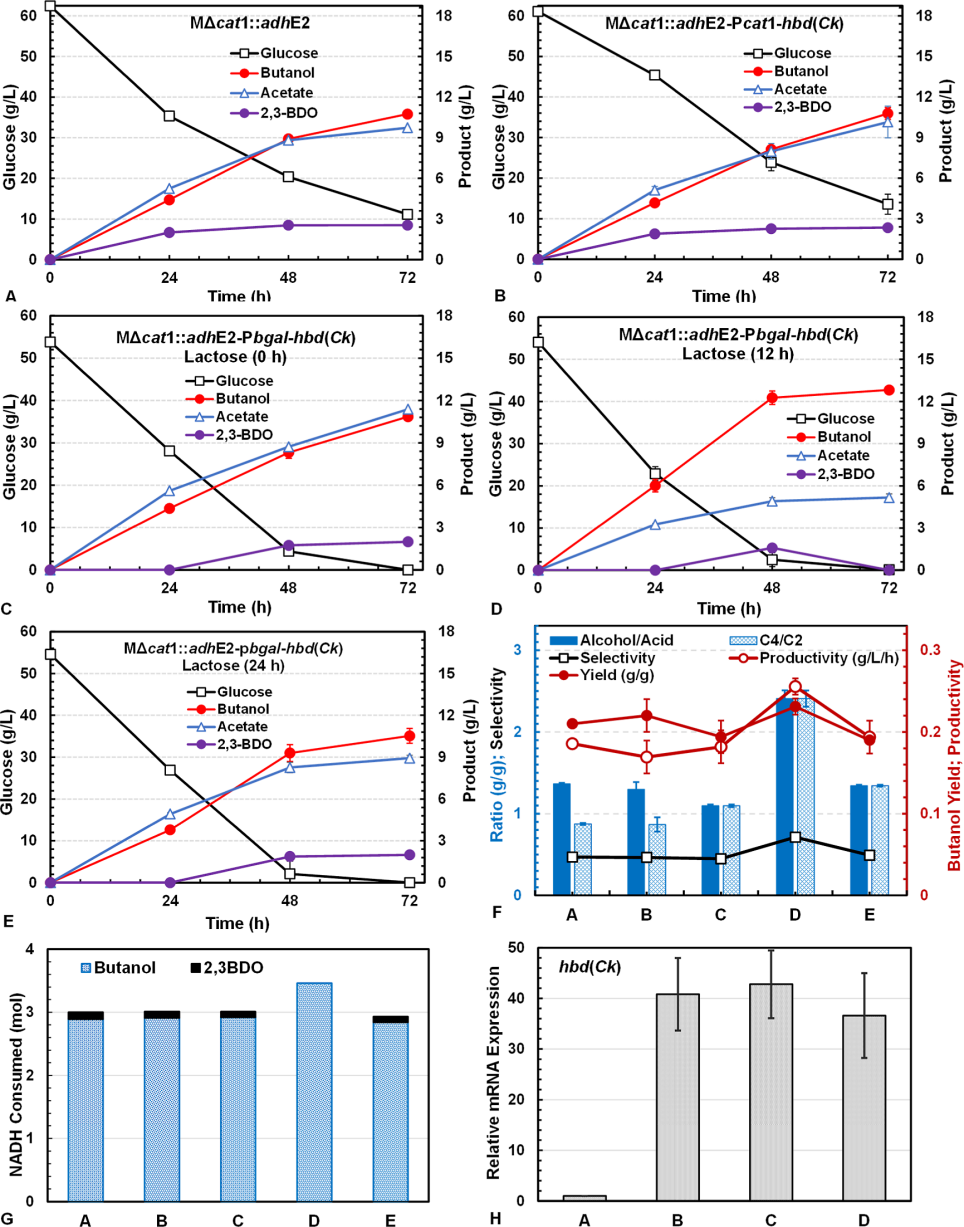
Batch fermentation kinetics of *C. tyrobutyricum* MΔ*cat*1::*adh*E2 (A), MΔ*cat*1::*adh*E2-P*cat*1-*hbd*(*Ck*) (B), and MΔ*cat*1::*adh*E2-P*bgal*-*hbd*(*Ck*) with *hbd* expression induced by lactose at 0 h (C), 12 h (D), and 24 h (E), respectively, in serum bottles; the butanol yield, productivity, selectivity, and alcohol/acid and C4/C2 ratios (F); NADH consumption associated with butanol and 2,3-BDO biosynthesis per mol of glucose consumed in the fermentations (G); and the relative transcriptional (mRNA) levels of *hbd*(*Ck*) in cells at 36 h in the batch cultures (H). Butanol productivity was based on data at 48 h, selectivity and C4/C2 and alcohol/acid ratios were based on the end points, butanol yield was estimated from the linear regression of butanol vs glucose in the entire batch fermentation, and NADH consumptions (mol/mol glucose consumed) associated with each product biosynthesis was calculated from the product yield using the stoichiometric equations given in Table S2. Relative *hbd*(*Ck*) transcription levels were determined in qRT-PCR with *typ*A as the internal reference gene. All experiments were conducted in biological duplicate with the average and standard deviation (error bar) reported in the figures. Trace amounts of ethanol (<0.5 g/L) were also detected in GC analysis but are not included in the fermentation time course figures. Their impacts on the fermentation flux calculation would be small and can be neglected.

Finally, the relative *hbd*(*Ck*) transcription levels in cells overexpressing *hbd*(*Ck*) (vs control without the overexpressing *hbd*(*Ck*) gene) were estimated using qRT-PCR with *typ*A as the internal reference gene. The results confirmed robust *hbd*(*Ck*) transcription in cells with 30- to 40-fold higher than the nonexpression control (MΔ*cat*1::*adh*E2) (Figure 1H). The P*bgal* promoter induced with 40 mM lactose at 0 and 12 h gave mRNA transcription levels similar to that from the constitutive P*cat*1 promoter, confirming that the improved phenotype of MΔ*cat*1::*adh*E2-P*bgal*-*hbd*(*Ck*) was attributable to optimized induction timing rather than a different *hbd*(*Ck*) expression level. It should be noted that P*bgal* induction with lactose at 20, 40, and 80 mM, respectively, gave similar fermentation performance, although the *hbd*(*Ck*) mRNA expression level increased with increasing lactose concentration (Figure S3). Therefore, 40 mM lactose was used in all of the subsequent experiments in this study.

It can be concluded that optimal induction timing (at 12 h) allowed cells to use NAD^+^ for glycolysis and NADPH for anabolism in the exponential phase and then upon inducer addition to use NADPH for the HBD reaction and NADH for butanol biosynthesis, thus meeting both cell growth and butanol production needs. These findings confirm that fine-tuning the timing of *hbd*(*Ck*) expression is critical for balancing metabolic flux and redox cofactors, which in turn minimizes acetate production and results in higher butanol production. Fine-tuning the induction timing and expression levels of exogenous enzymes in engineered pathways has been used to minimize the metabolic burden and relieve the bottlenecks in the reactions, leading to the overproduction of targeted metabolites in recombinant microorganisms.^29,30^ However, this study, for the first time, demonstrated that optimizing the induction time for *hbd*(*Ck*) expression significantly enhanced butanol production in *C. tyrobutyricum* MΔ*cat*1::*adh*E2-P*bgal*-*hbd*(*Ck*), which did not produce butyrate because of the knockout of *cat*1 encoding for the CoA transferase responsible for the assimilation of acetate and biosynthesis of butyrate in *C. tyrobutyricum*.^31^

### 2.2 Effects of hbd­(Ck) Expression on Cell Growth

The intracellular concentrations of NAD­(P)­H/NAD­(P)^+^ play a critical role in regulating bacterial growth and metabolism.^27^ NAD^+^ is an essential electron acceptor in glycolysis, and NADH is widely used as an electron donor in metabolism and the electron transport system to generate ATP needed for cell growth and maintenance, whereas NADPH is an electron donor in the biosynthesis of essential biomolecules (fatty acids, amino acids, nucleotides, etc.).^32^ We hypothesized that overexpression of NADPH-dependent HBD in *C. tyrobutyricum* Δ*cat*1::*adh*E2 might disrupt its NADH/NAD^+^ homeostasis, resulting in inefficient glycolysis and poor cell growth and metabolic activities. The effects of *hbd*(*Ck*) expression on the growth kinetics of MΔ*cat*1::*adh*E2 and its derivative strains expressing *hbd*(*Ck*) under P*cat*1 or P*bgal* control, respectively, were thus investigated in serum tubes with cell density monitored as the optical density (OD_600_) in batch cultures. Figure 2 shows the growth kinetics and compares the specific growth rates for the various strains and conditions studied. In general, cell growth was inhibited by the *hbd*(*Ck*) expression with a significantly decreased specific growth rate in the exponential phase and lowered stationary cell density for MΔ*cat*1::*adh*E2-P*cat*1-*hbd*(*Ck*), compared to MΔ*cat*1::*adh*E2, and MΔ*cat*1::*adh*E2-P*bgal*-*hbd*(*Ck*), with lactose induction at 0 h, compared to at 12 h or no lactose induction. The results confirmed that optimized induction timing of *hbd*(*Ck*) alleviated the adverse effects of *hbd*(*Ck*) overexpression on cell growth and was able to maintain stable redox homeostasis in cells, which is essential for efficient production of metabolites such as butanol in industrial fermentation.^33–35^

**Figure 2:**
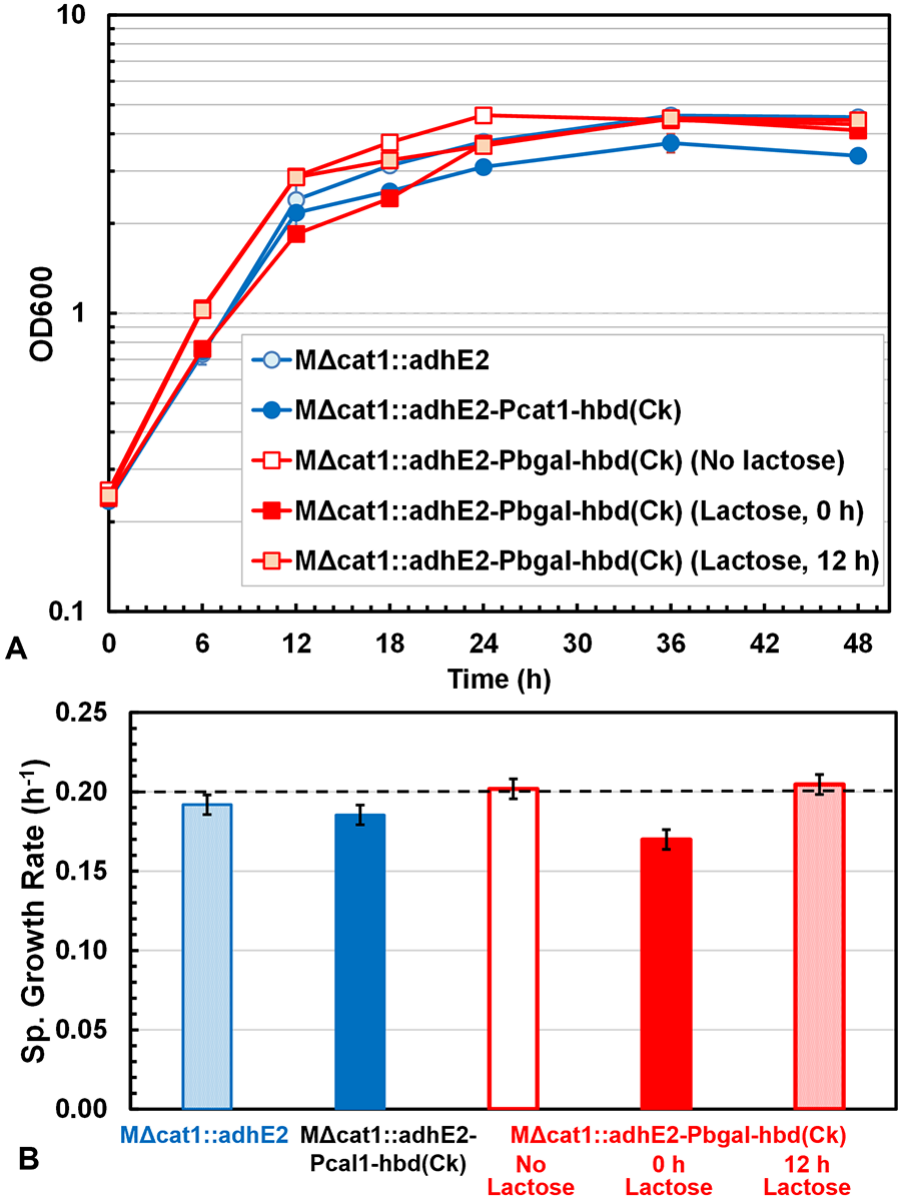
Effects of *hbd*(*Ck*) expression on the growth kinetics of MΔ*cat*1::*adh*E2 and its derivative strains expressing *hbd*(*Ck*) under P*cat*1 or P*bgal* control, respectively. Lactose (40 mM) was added at 0 or 12 h for MΔ*cat*1::*adh*E2-P*bgal*-*hbd*(*Ck*) to evaluate the *hbd*(*Ck*) effect on growth. (A) Growth curves during 48 h cultivation in 20 mL serum tubes with cell density monitored as OD_600_. (B) Comparison of the specific growth rates in the exponential phase estimated from the OD data in the first 12 h. Cell growth was significantly decreased and reached a lower stationary cell density for MΔ*cat*1::*adh*E2-P*cat*1-*hbd*(*Ck*) compared to MΔ*cat*1::*adh*E2. Similarly, growth was slower for MΔ*cat*1::*adh*E2-P*bgal*-*hbd*(*Ck*) with lactose induction at 0 h compared to at 12 h or no lactose induction.

### 2.3 Effects of MV Addition

As an artificial electron carrier, MV can divert electrons for hydrogen production to NADH regeneration, thereby increasing the intracellular NADH pool for butanol biosynthesis.^8,18,36^ Therefore, MV addition at various concentrations up to 200 μM was investigated for its effect on butanol production by MΔ*cat*1::*adh*E2-P*bgal*-*hbd*(*Ck*) in batch fermentation (Figure 3). However, no significant difference in butanol production was observed among all MV concentrations (0, 50, 100, and 200 μM) studied. In general, most of the glucose was rapidly consumed in 48 h, coupled with rapid production of butanol at all MV concentrations. The fermentation ceased at 96 h when the butanol concentration reached ∼17.5 g/L with little glucose left. Although MV addition did decrease acetate production from 5 g/L at MV0 to 3 g/L at MV200, lactate production increased to 2 g/L at MV200. Consequently, butanol yield from glucose remained unchanged at 0.283 ± 0.005 g/g for all MV concentrations studied (Figure 3E). Nevertheless, the product selectivity increased from 0.70 without MV (MV0) to 0.78 with 100 μM MV. The butanol productivity from these fermentations was 0.32 ± 0.01 g/L/h. About 2.75 mol or 69% of total reducing equivalents (NADH + reduced ferredoxin, Fd_re_) generated from each mole of glucose consumed in glycolysis was used in butanol biosynthesis (Figure 3F). The inducible *hbd*(*Ck*) expression at 12 h was effective in providing NADH needed for butanol biosynthesis. Interestingly, butanol productivity in the fermentation increased slightly, instead of decreasing, with increasing MV concentration. These results are different from what have been observed with WT-*adh*E2, Ack-*adh*E2, and Δ*cat*1::*adh*E2 that butanol yield increased while productivity decreased with increasing MV concentration.^18,26^

**Figure 3:**
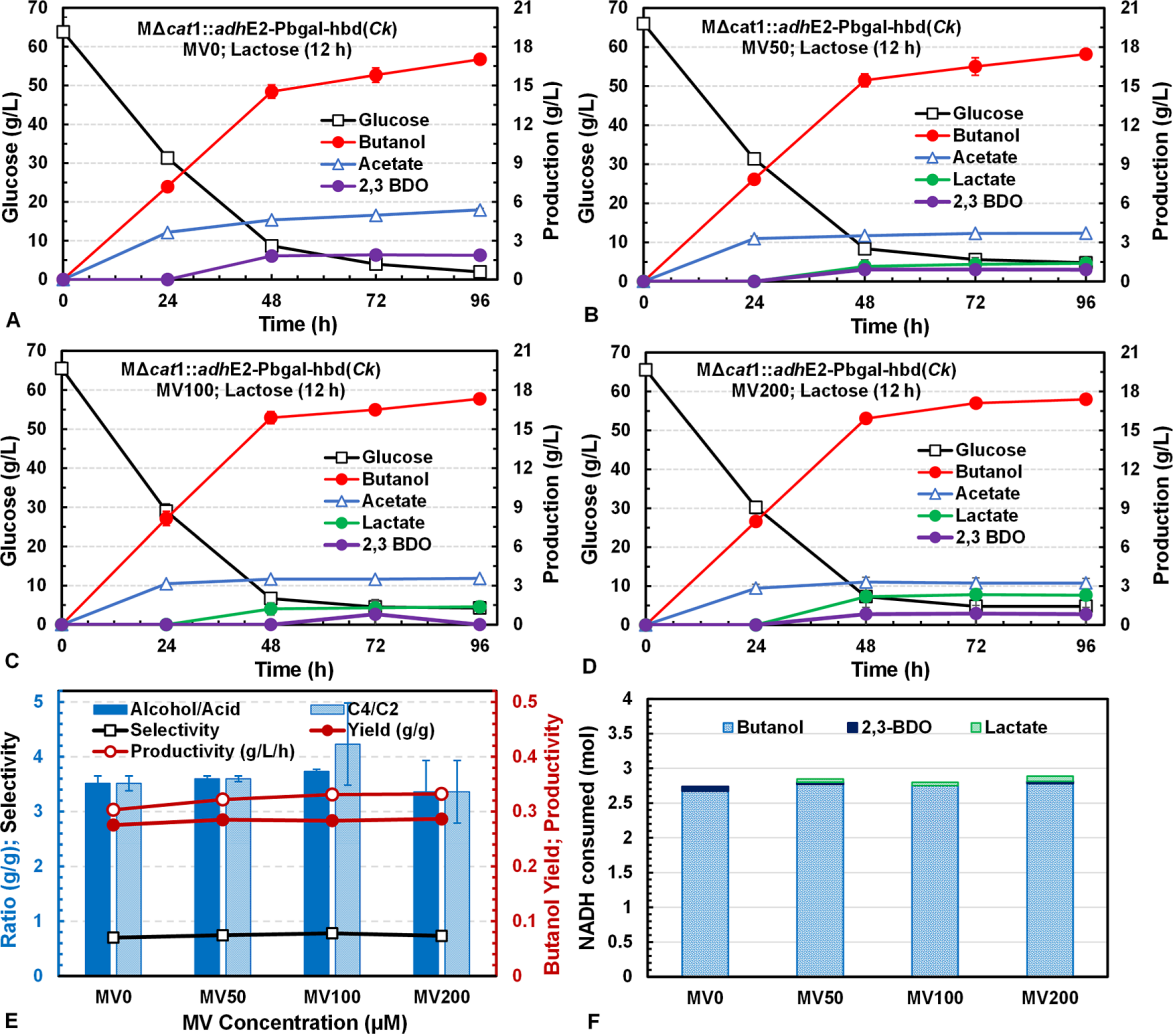
Batch fermentations by *C. tyrobutyricum* MΔ*cat*1::*adh*E2-*hbd*(*Ck*) with *hbd* expression induced by lactose at 12 h in serum bottles with methyl viologen (MV) at various concentrations also added at 12 h: (A) 0 μM MV; (B) 50 μM MV; (C) 100 μM MV; and (D) 200 μM MV. (E) C4/C2 and alcohol/acid ratios, selectivity, and butanol yield and productivity based on the data at 48 h. (F) NADH consumption associated with butanol, lactate, and 2,3-BDO biosynthesis per mol of glucose consumed in the fermentations.

The results imply that inducible expression of *hbd*(*Ck*) can be used as an effective strategy for increasing the NADH pool without the MV addition. To boost NADH availability for butanol biosynthesis, prior studies have focused on NADH regeneration strategies, such as adding MV^18^ and benzyl viologen,^37^ introducing the exogenous formate dehydrogenase^38^ and ferredoxin-NAD­(P^+^) oxidoreductase,^39^ and integrating bioelectrochemical systems or a photocatalyst to supply electrons to the culture.^40,41^ Alternatively, NADH availability for butanol biosynthesis can be increased through reducing NADH consumption by replacing the NADH-dependent HBD with NADPH-dependent HBD in the metabolic pathway.^26^ However, constitutive expression of NADPH-dependent HBD might cause growth inhibition due to NADH/NADPH imbalance, which was mitigated through the inducible expression of *hbd*(*Ck*) at an optimal induction time during batch culture without adding MV. The internal genetic regulation of redox reactions avoids potential toxicity and process complexity and is preferable to chemical redox mediation and bioelectrochemical systems.

### 2.4 Effects of Low Temperature

Decreasing culture temperature can reduce membrane fluidity and permeability and thus improve the cell’s solvent tolerance.^23,42^ We evaluated the effects of low temperature (25 °C) on the batch culture of MΔ*cat*1::*adh*E2-P*bgal*-*hbd*(*Ck*) with *hbd*(*Ck*) induced at 12 h (Figure 4). As expected, the low-temperature culture improved butanol production to reach a high titer of 23 g/L at 144 h, accompanied by small amounts of acetate (2.1 g/L) and 2,3-BDO (2.1 g/L; Figure 4A). Adding 50 μM MV in the low-temperature culture decreased acetate (0.4 g/L) and 2,3-BDO (1.8 g/L) production, but butyrate production increased to 1.5 g/L, while butanol production did not change (Figure 4B). The production of butyrate in these fermentations suggested the presence of functional thioesterases, such as the one encoded by *cat*2 (CTK_RS03720),^31^ in the genome of *C. tyrobutyricum*, which remains to be verified. Increasing glucose in the medium to 120 g/L did not raise the final butanol titer above 23 g/L even though the fermentation time was extended to 168 h (Figure 4C,D). Apparently, 23 g/L butanol was the tolerance limit for *C. tyrobutyricum* at 25 °C. The low-temperature cultures also gave a much higher butanol yield (0.31 ± 0.01 g/g glucose vs 0.28 ± 0.01 g/g glucose at 37 °C) and product selectivity (∼0.85 vs ∼0.72 at 37 °C) (Figure 4E). About 3 mol or 75% of the reducing equivalents (NADH + Fd_re_) generated from each mole of glucose consumed in glycolysis was used in butanol biosynthesis (Figure 4F). However, the butanol productivity at 25 °C was only ∼0.16 g/L·h or 50% of that at 37 °C. MFA showed that at 25 °C, 51% of the substrate carbon was retained in butanol and only 2.9% in acetate, whereas at 37 °C, 44% of the substrate carbon was retained in butanol and 3.1% in acetate (see Figure S2).

**Figure 4:**
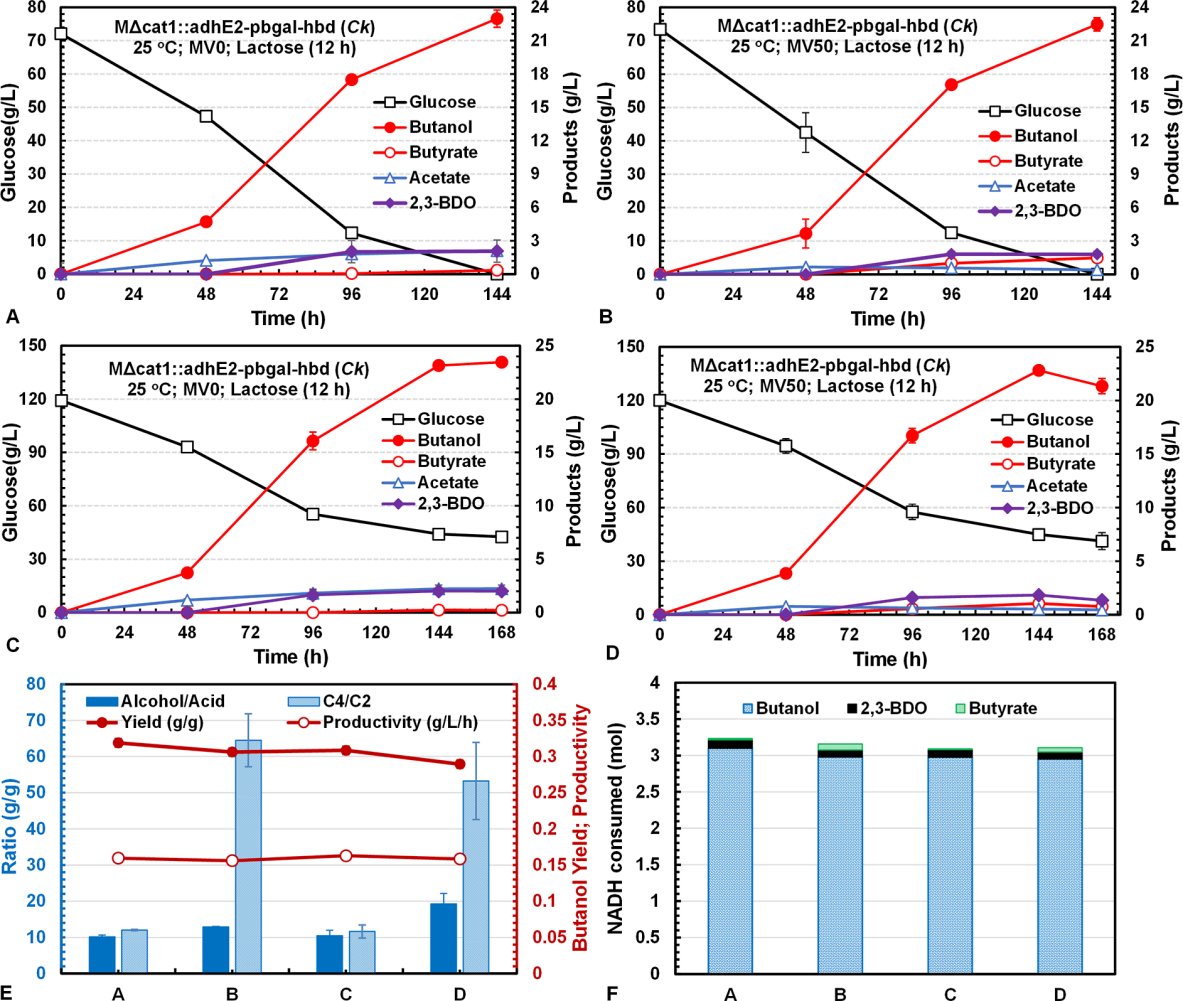
Batch fermentations by *C. tyrobutyricum* MΔ*cat*1::*adh*E2-*hbd*(*Ck*) with *hbd* expression induced by lactose at 12 h in serum bottles at 25 °C: (A) 0 μM MV with initial glucose 70 g/L; (B) 50 μM MV with initial glucose 70 g/L; (C) 0 μM MV with initial glucose 120 g/L; (D) 50 μM MV with initial glucose 120 g/L; (E) C4/C2 and alcohol/acid ratios and butanol yield and productivity; and (F) NADH consumption associated with butanol, butyrate, and 2,3-BDO biosynthesis per mol of glucose consumed in the fermentations.

These results confirm that low-temperature fermentation (25 °C) can effectively increase butanol production to achieve a high titer (23 g/L) and yield (0.31 g/g) due to enhanced butanol tolerance. Lowering the fermentation temperature to 20 °C could further increase the butanol titer to 26 g/L, as previously reported for Δ*cat*1::*adh*E2,^23^ but the productivity would be much lower. It is noted that the butanol yield from Δ*cat*1::*adh*E2 was much lower at 0.23 g/g due to the excessive amounts of acetate and ethanol coproduced with butanol. Similarly, *Clostridium carboxidivorans* produced more butanol and ethanol but at significantly reduced rates at 25 °C, while 37 °C provided the optimal conditions for cell growth and organic acids production.^42^ Although lowering the fermentation temperature could greatly increase the product titer and yield, the productivity is severely compromised. Cell immobilization or cell recycling in a continuous or sequential batch fermentation process may be used to greatly increase cell density in the bioreactor and thus the reactor productivity, as demonstrated in previous studies.^10,19,20,43^

### 2.5 Batch Fermentation in the Bioreactor

To scale up the butanol fermentation for industrial applications, we evaluated MΔ*cat*1::*adh*E2-P*bgal*-*hbd*(*Ck*) in a stirred-tank bioreactor operated at 37 °C with pH controlled at 5.5 by using NH_4_OH, which allowed us to monitor cell growth by OD measurements that could not be done in serum-bottle fermentations with CaCO_3_ for pH buffer. Lactose was added to induce the expression of *hbd*(*Ck*) when the cell density or OD reached a predetermined level (0, 2.3, 4.1, 6.5, and 8.3) corresponding to early and mid-exponential phases in the batch culture (Figure 5). In general, the fermentation kinetics in glucose consumption and butanol and acetate production were similar to those observed in serum bottles. When lactose was added at the beginning or before inoculation (OD0), there was an apparent lag phase of ∼12 h with negligible cell growth and slow glucose consumption, followed by accelerated glucose consumption and butanol production reaching 18.1 g/L at 72 h (Figure 5A). The corresponding butanol yield of 0.27 g/g and productivity of 0.28 g/L·h were much higher than those obtained in serum bottles with lactose addition at 0 h. The improvements might be attributed to the NH_4_OH used in controlling the bioreactor pH as adequate amounts of ammonia could stimulate cell growth by enhancing the nucleic acid and protein synthesis.^44,45^ Meanwhile, a slightly delayed expression of *hbd*(*Ck*) in the early exponential phase after OD had reached 2.3 allowed cells to grow without an apparent lag phase and increased butanol production to the highest titer, productivity, and yield (21.7 g/L, 0.38 g/L·h, and 0.30 g/g, respectively) at 72 h (Figure 5B). On the other hand, lactose addition at higher ODs (4.1, 6.5, and 8.3) or in the mid exponential phase led to a progressive decrease in butanol production (Figure 5C–E) and product selectivity (Figure 5F). The cultures with delayed *hbd* (*Ck*) induction at OD 6.5 and 8.3 also showed significantly slowed glucose consumption or metabolism, probably caused by an imbalance in NADH/NADPH with the overexpression of NADP-dependent HBD. Induction at OD 2.3 gave the highest butanol production in titer, productivity, and yield (Figure 5F). These results suggested that inducible expression of *hbd*(*Ck*) at OD 2.3 or in the early exponential phase provided the optimal balance between cell growth and redox metabolism favoring butanol production in *C. tyrobutyricum* MΔ*cat*1::*adh*E2-P*bgal*-*hbd*(*Ck*).

**Figure 5:**
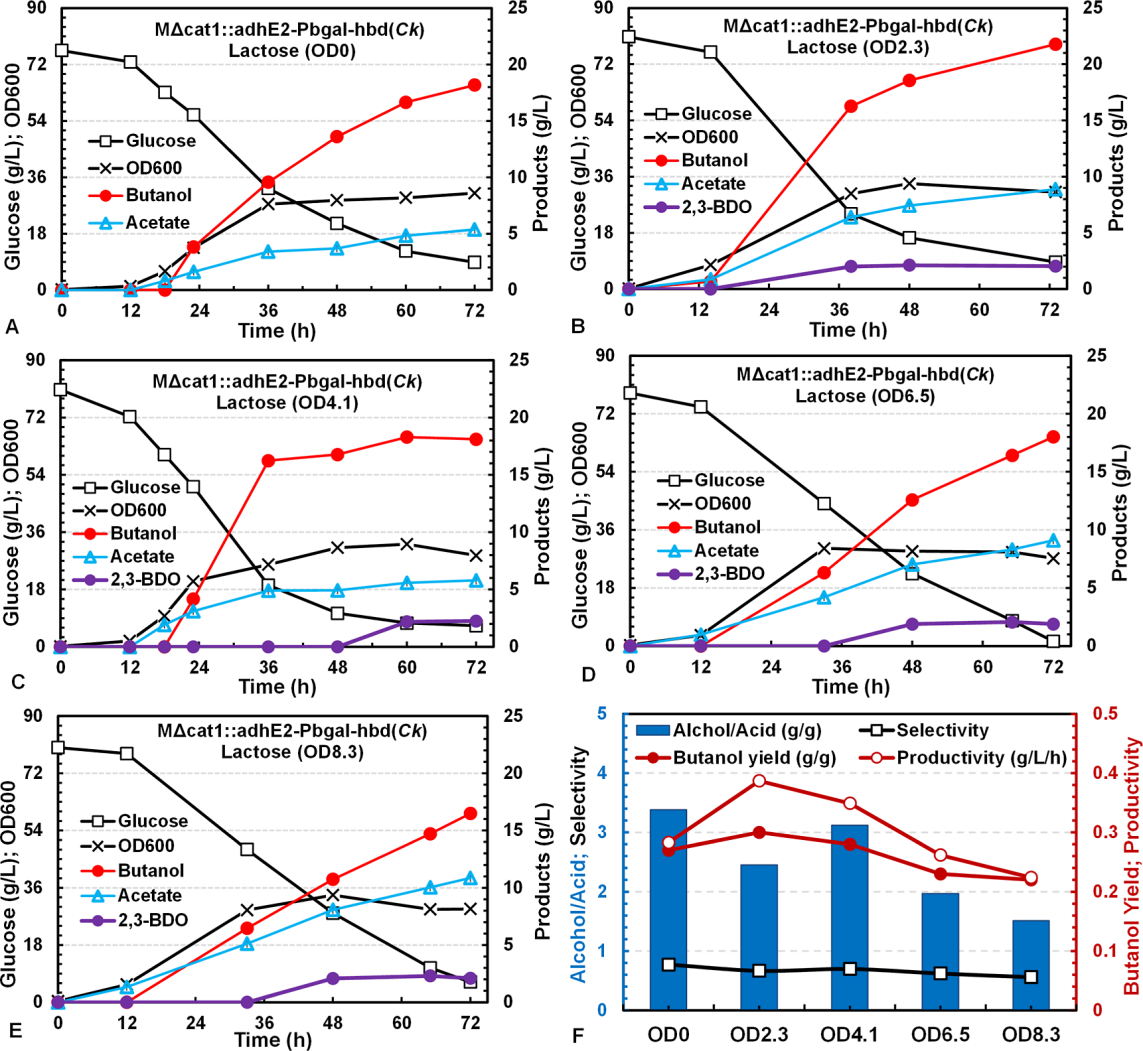
Batch fermentation kinetics of *C. tyrobutyricum* MΔ*cat*1::*adh*E2-*hbd*(*Ck*) in bioreactors with *hbd* expression induced at OD0 (A), OD2.3 (B), OD4.1­(C), OD6.5 (D), and OD8.3 (E) and the corresponding alcohol/acid ratio, selectivity, and butanol yield and productivity (F).

### 2.6 Intracellular NAD­(P)H and NAD­(P)^+^

In *Clostridium*, NADH and NADPH serve distinct roles: NAD^+^/NADH drives catabolic energy generation and solvent production, whereas NADP^+^/NADPH primarily supports anabolic processes and biomass growth.^32,46,47^ The intracellular NADH/NAD^+^ ratio is a key factor affecting cell growth, glycolysis, and metabolic fluxes toward different metabolites. It is thus of high interest to know how dynamic *hbd*(Ck) expression affected redox metabolism and to elucidate why early exponential phase induction was optimal. We performed MFA and measured intracellular concentrations of NAD­(P)H and NAD­(P)^+^ at 18 and 36 h in cultures with *hbd*(Ck) expression induced immediately after inoculation (OD 0) and in the early exponential phase (OD 2.3) (Figure 6). Figure 6A shows the carbon flux distributions in key metabolic pathways calculated from the products (butanol, butyrate, acetate, ethanol, and 2,3-BDO) produced from glucose consumed in each of the batch fermentations in the bioreactor. When *hbd*(*Ck*) expression was induced in the early exponential phase at OD 2.3, ∼49% of the carbon present in the glucose consumed in the fermentation was channeled toward butanol biosynthesis and resulted in the highest butanol yield of 0.30 g/g, which could be attributed to the higher intracellular NADH/NAD^+^ pool, as evidenced by higher intracellular NADH/NAD^+^ levels at both 18 and 36 h compared with lactose addition at OD 0 (Figure 6B). This finding supports our hypothesis that the higher NADH availability with the early exponential phase induction gives higher butanol production. It should be noted that the NADH level was much lower, while NAD^+^ was much higher at 36 h than at 18 h, indicating that a significant amount of NADH was used for butanol biosynthesis. Interestingly, NADP^+^ and NADPH remained at a relatively low level throughout the fermentation in both cases, suggesting that the limited NADPH availability might have transiently limited cell growth. When NADPH-dependent HBD was overexpressed, cells must adjust their metabolism to generate the needed NADPH for anabolism to support cell growth.

**Figure 6:**
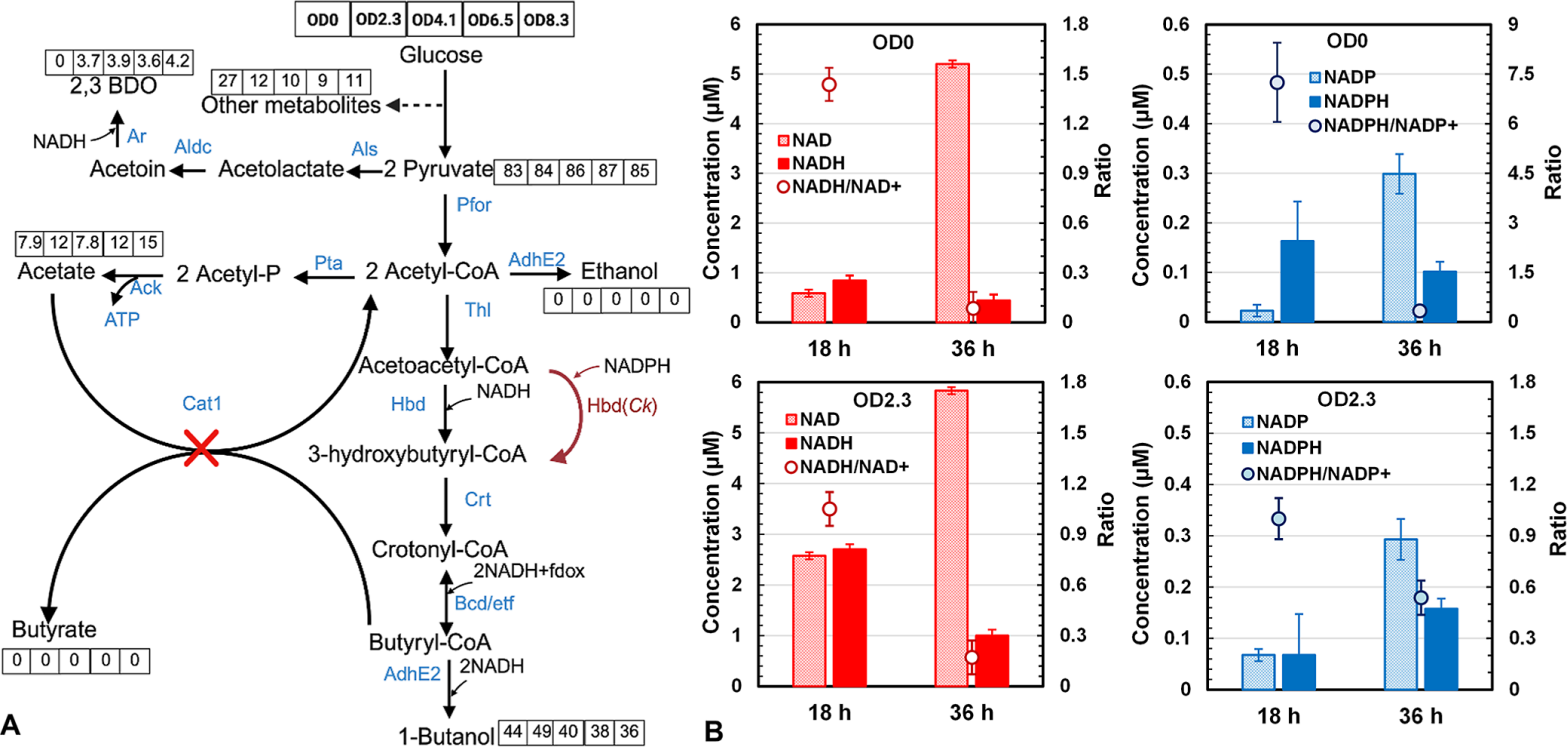
Effects of lactose addition at different OD levels on carbon flux distributions and intracellular NAD­(P)­H/NAD­(P)^+^ levels in *C. tyrobutyricum* MΔ*cat*1::*adh*E2-P*bgal*-*hbd*(*Ck*). (A) Metabolic flux distribution in key metabolic reactions. The flux values (%) given in the boxes next to the reactions are normalized with glucose (6 carbon) consumed in the fermentation being 100%. (B) Intracellular NAD­(P)­H/NAD­(P)^+^ levels at 18 and 36 h in batch cultures with *hbd*(*Ck*) induction at OD0 and OD2.3.

These findings explain why the induction expression of *hbd*(Ck) at an optimal time is important to butanol production in *C. tyrobutyricum* MΔ*cat*1::*adh*E2-P*bgal*-*hbd*(*Ck*). Introducing the heterologous enzyme (HBD) activity “just-in-time” when the metabolic state shifted from growth to metabolite (butanol) production was critical to the production of the metabolite in fermentation.^48,49^ Overall, the dynamic expression strategy balanced the intracellular NADH/NAD^+^ and NADPH/NADP^+^ more effectively than the constitutive expression strategy, allowing cells to match cofactor availability with phase-specific metabolic needs.

## 3 Discussion

Classical ABE fermentation with solventogenic clostridia coproduces butanol, acetone, and ethanol at a 6:3:1 ratio, with butanol production TRY typically at ∼12 g/L, ∼0.35 g/L·h, and ∼0.2 g/g glucose consumed.^8,50–52^ Extensive engineering of *C. acetobutylicum* to eliminate byproducts acetone, by knocking out *ctf*AB (CoA transferase), and butyrate, by knocking out *ptb* (phosphotransbutyrylase) and *buk* (butyrate kinase), and to increase NADH availability, by knocking out the redox-sensing transcriptional regulator *rex* that represses *thl*A, *hbd*, *crt*, and *bcd-etf*AB in the C4 formation pathway (from acetyl-CoA to butyryl-CoA) and *adh*E2, greatly increased butanol yield to ∼0.32 g/g or ∼80% of the theoretical maximum butanol yield, but the butanol titer was only ∼12 g/L.^24^ Earlier studies also demonstrated that overexpressing thiolase (*thl*A) improved butanol production in *C. acetobutylicum* and *C. cellulovorans*.^25,53,54^ Expressing a heterologous pathway comprising four genes (*ato*B from *Escherichia coli*, *hbd* from *C. kluyveri*, *crt* from *C. acetobutylicum,* and *ter* from *Treponema denticola*) in engineered *E. coli* produced butanol at 15 g/L with a yield of >70% of the theoretical yield, whereas less than1 g/L butanol was produced by engineered *E. coli* overexpressing clostridial genes *thl*A, *hbd*, *crt*, and *bcd-etf*AB.^55^ This was because the *E. coli* thiolase encoded by atoB could tolerate a higher intracellular CoA level and would pull more carbon flux from acetyl-CoA into acetoacetyl-CoA^55–57^ and the enzyme encoded by *ter* from *T. denticola* replacing BCD-ETF complex catalyzed the reaction irreversibly.^58,59^ These two genes together greatly boosted the metabolic flux from C2 acetyl-CoA to C4 butyryl-CoA and thus enhanced butanol production in engineered *E. coli*.

Our present study extends previous metabolic engineering efforts in several important ways by achieving a unique balance between cell growth and redox metabolism in MΔ*cat*1::*adh*E2-P*bgal*-*hbd*(*Ck*) to attain the highest butanol TRY (>20 g/L, >0.35 g/L·h, >0.30 g/g) and selectivity (butanol/all metabolic products >0.80), which compare favorably with native and engineered microorganisms including *E. coli*, *C. acetobutylicum*, and *C. tyrobutyricum* Δ*cat*1::*adh*E2 and Δ*cat*1::*adh*E2-P*cat*1-*hbd*(*Ck*) (see Table 1). The higher butanol titer can be attributed to the high butanol tolerance of *C. tyrobutyricum*,^15^ whereas the higher butanol yield can be attributed to the high product selectivity (>0.8) due to much reduced acetate and ethanol production via the inducible *hbd*(*Ck*) expression in *C. tyrobutyricum* with Δ*cat*1 that could eliminate butyrate biosynthesis.^23,26^ Moreover, the high butanol titer (21.8 g/L), yield (0.30 g/g), and productivity (0.39 g/L·h) obtained in the stirred-tank bioreactor at 37 °C also compared favorably with those obtained with Ack-*adh*E2 in repeated batch fermentation with cells immobilized in a fibrous bed bioreactor (FBB) and with the addition of 250 μM MV,^19^ which greatly increased butanol production because of the high-cell-density culture environment and the enhancement effect of MV.^18^ It is thus possible to further increase butanol production to reach a much higher TRY using MΔ*cat*1::*adh*E2-P*bgal*-*hbd*(*Ck*) in FBB fermentation. Compared to industrial ABE fermentation, butanol titer increased >66% (from 12 g/L to >20 g/L), which would reduce energy cost by >40% for butanol separation, and butanol yield increased >50% (from 0.2 g/g to >0.30 g/g), which would reduce feedstock cost by >33%. Therefore, the improvements in TRY with the inducible *hbd*(*Ck*) expression in MΔ*cat*1::*adh*E2-P*bgal*-*hbd*(*Ck*) should significantly reduce the butanol production cost from the current $4.50 per gallon using the ABE fermentation process to less than $2.50 per gallon, which would be highly competitive to bioethanol and petroleum-derived butanol.^19^

**Table 1:** Comparison of Butanol Yield, Productivity, Selectivity, and Alcohol/Acid and C4/C2 Ratios in Batch Fermentations with Various *C. tyrobutyricum* Strains^a^

strains	temp (°C)	culture system	MV (μM)	butanol yield (g/g)	productivity (g/L·h)	selectivity (g/g)	alcohol/acid (g/g)	C4/C2 (g/g)	ref
Δ*cat*1::*adh*E2	37	serum bottle	0	0.21	0.16	0.50	1.21	1.21	^26^
	37	bioreactor	0	0.20	0.31	0.41	1.27	0.77	^23^
	25	bioreactor	0	0.23	0.21	0.47	2.43	1.02	
Δ*cat*1::*adh*E2-P*cat*1-*hbd* (*Ck*)	37	serum bottle	0	0.21	0.13	0.50	1.32	1.08	^26^
	37	serum bottle	50	0.29	0.22	0.73	4.69	3.75	
MΔ*cat*1::*adh*E2	37	serum bottle	0	0.21 ± 0.01	0.19 ± 0.01	0.47 ± 0.01	1.37	0.88	this study
MΔcat1::adhE2-Pcat1-hbd(*Ck*)	37	serum bottle	0	0.22 ± 0.01	0.17 ± 0.01	0.46 ± 0.01	1.30	0.87	
MΔ*cat*1::*adh*E2-P*bgal*-*hbd* (*Ck*)	37	serum bottle	0	0.27 ± 0.01	0.30 ± 0.01	0.70 ± 0.01	3.51	3.51	this study
	37	serum bottle	50	0.28 ± 0.01	0.32 ± 0.01	0.74 ± 0.05	3.59	3.59	
	37	serum bottle	100	0.28 ± 0.01	0.33 ± 0.01	0.77 ± 0.01	3.73	4.22	
	37	serum bottle	200	0.28 ± 0.01	0.33 ± 0.01	0.73 ± 0.08	3.36	3.36	
	25	serum bottle	0	0.32 ± 0.01	0.16 ± 0.01	0.83 ± 0.01	10.5	12.0	
	25	serum bottle	50	0.31 ± 0.01	0.16 ± 0.01	0.86 ± 0.01	12.9	32.3	
	37	bioreactor	0	0.30	0.38	0.67	2.45	2.45	
WT-*adh*E2-*hbd* (*Ck*)	37	bioreactor	0	0.22	0.31	0.46	0.91	9.31	^26^
Ack-*adh*E2-*hbd* (*Ck*)	37	bioreactor	0	0.25	0.35	0.44	1.26	8.03	
Ack-adhE2	37	FBB	250	0.28–0.32	0.18–0.34	0.65–0.69	1.9–2.2	70	^19^

a P*bgal* promoter was induced with 40 mM lactose at 12 h in batch fermentations with MΔ*cat*1:*adh*E2-P*bgal*-*hbd*(*Ck*); FBB: fibrous bed bioreactor.

Cofactor engineering has been widely used as an effective strategy for regulating redox homeostasis, relieving metabolic burden to improve robustness of cells, and increasing bioproduction of chemicals and biofuels in fermentation.^33–35^ For example, altering the intracellular redox ratio through the expression of a heterologous NADH oxidase in *E. coli* reduced the NADH/NAD^+^ ratio, which alleviated acetate overflow under high glucose uptake conditions.^60^ Also, changing the NADH/NAD^+^ ratio by overexpressing *pnc*B in *E. coli* redirected the metabolic flux from lactate to ethanol biosynthesis.^61^ Furthermore, the nisin-inducible expression of a heterologous NADH oxidase in *Lactococcus lactis* decreased the intracellular NADH/NAD^+^ ratio and caused a marked redistribution of pyruvate flux away from lactate and toward mixed-acid products.^62^ The xylitol biosynthesis from xylose in an engineered *E. coli* was boosted by dynamically regulating the NADPH-consuming enoyl-ACP reductase and NADPH-producing glucose-6-phosphate dehydrogenase to improve NADPH flux using a two-stage gene circuit.^63^ In our study, we also demonstrated the dynamic regulation of NAD­(P)­H/NAD­(P)^+^ through the timely induction of *hbd*(*Ck*) expression using an inducible promoter for butanol production by engineered *C. tyrobutyricum*. Overall, dynamic gene expression is highly effective in balancing intracellular NADH/NAD^+^ and NADPH/NADP^+^ and allows the mutant to match cofactor availability with phase-specific metabolic needs during fermentation. Although gene expression can also be modulated by using constitutive promoters with balanced transcriptional strength, it is cumbersome and labor-intensive to screen promoters with an appropriate strength and building a gene circuit with a biosensor to switch on or off the promoter.^64^

In summary, *C. tyrobutyricum* MΔ*cat*1::*adh*E2-P*bgal*-*hbd*(*Ck*) with inducible expression of *hbd*(*Ck*) significantly enhanced butanol production TRY and selectivity. Although the current butanol yield of 0.32 g/g (∼80% of the theoretical value 0.41 g/g) achieved in this study is among the highest ever reported for a biobutanol fermentation process, it may be further increased with *rex* knockout and expression of heterologous *ato*B and *ter*, as demonstrated in recombinant *C. acetobutylicum* and *E. coli*.^24,55^ It should be noted that small but significant amounts of lactate and 2,3-BDO were also produced by MΔ*cat*1::*adh*E2-P*bgal*-*hbd*(*Ck*) in fermentation, which not only consumed some NADH but also reduced the butanol yield from glucose. It is thus desirable to knock out genes in the lactate and 2,3-butanediol biosynthesis pathways (see Figure S1) to further increase butanol yield. Meanwhile, protein engineering via active-site mutagenesis can be applied to the enzyme AAD (encoded by *adh*E2) to increase its activity and selectivity for the conversion of butyryl-CoA to butyraldehyde and butanol, which could be a bottleneck in butanol biosynthesis.^65^ Finally, the persistent deficiency in the NADPH supply, as indicated by low intracellular NADPH/NADP^+^ levels, may also limit the fermentation. Engineering NADH-dependent variants of HBD and enhancing NADPH-generating pathways (e.g., the pentose phosphate pathway) could alleviate the redox imbalance caused by the overexpression of NADPH-dependent HBD and allow for constitutive or earlier induction without compromising cell growth and catabolism. Overexpressing a heterologous NAD^+^ kinase, which catalyzes the phosphorylation of NAD^+^ to NADP^+^,^66^ may increase the intracellular NADP^+^/NADPH pool and offset the adverse effect of *hbd*(*Ck*) overexpression. In addition, the Rex regulator plays a vital role in sensing redox homeostasis and represses *bcs* operon genes involved in the metabolic reactions from acetyl-CoA to butyryl-CoA when the NAD^+^/NADH ratio is high.^67^ These should be considered in the further development of an ideal biobutanol cell factory.

In conclusion, dynamically controlling the expression of *hbd*(*Ck*) in *C. tyrobutyricum* MΔ*cat*1::*adh*E2-P*bgal*-*hbd*(*Ck*) via lactose induction in the early exponential phase alleviated redox imbalance and growth inhibition caused by the constitutive expression of NADPH-dependent HBD and gave the highest butanol production TRY (21.8 g/L, 0.39 g/L·h, 0.30 g/g) and selectivity (>0.7) in batch fermentation in a bioreactor operated at 37 °C and pH 5.5. At a lower temperature (25 °C), butanol production in the fermentation increased to 23 g/L and 0.32 g/g with >0.85 product selectivity due to much reduced byproducts formation, but productivity decreased to 0.16 g/L·h. The fermentation using the engineered *C. tyrobutyricum* strain offers a scalable and economically viable biobutanol production route for industrial application.

## 4 Materials and Methods

### 4.1 Strains and Culture Media

*E. coli* DH5α and CA434 (donor cells in conjugation) were grown at 37 °C in LB medium containing chloramphenicol. Unless otherwise noted, *C. tyrobutyricum* ATCC 25755 (wild type, WT) and its mutant strains were cultured anaerobically at 37 °C in RCM containing antibiotics.^26^ All serum-bottle fermentation kinetic studies were conducted in CGM containing 50–120 g/L glucose and 40 g/L CaCO_3_ for buffering the culture at ∼pH 5.5.

### 4.2 Recombinant Plasmids and Mutant Strains

Table 2 lists the recombinant plasmids and strains used and constructed in this study. The gene knockout plasmid was constructed using an endogenous CRISPR-Cas system, with backbones derived from pMTL83151 and pMTL85151.^16,23,68^ First, pMTL83151 (or pMTL85151) was linearized. Next, the lactose inducible promoter and terminator (a 30 bp repeat sequence) were amplified and connected by overlapping PCR. We then amplified the 1000 bp homologous arm from *C. tyrobutyricum* ATCC 25755. These fragments were ligated with the linearized vector to yield pMTL83151Δ*rease*, pMTL85151ΔI, pMTL85151Δ*upp*, and pMTL83153Δ*cat*1::*adh*E2, respectively. Overexpressing plasmids were built on the pMTL82151 containing the lactose inducible promoter P*bgal*.^30^

**Table 2:** Mutants and Plasmids Used in This Study

strain/plasmids	description/characteristics
strains	
WT	Clostridium tyrobutyricum ATCC 25755
M	C. tyrobutyricum with the inactivation of restriction modification system (Rease; CTK_RS13400), native plasmid (I; pCTK01) and *upp* (CTK_RS00780) knockouts using the CRSPR-Cas plasmids pMTL83151Δ*rease*, pMTL85151ΔI, and pMTL85151Δ*upp*, respectively
Δ*cat*1::*adh*E2	WT with the *cat*1 knockout and *adh*E2 knock-in on the genome using pMTL82151-Δ*cat*1::*adh*E2
Δ*cat*1::*adh*E2-P*cat*1-*hbd* (*Ck*)	Δ*cat*1::*adh*E2 transformed with pMTL82151-P*cat*1-*hbd* (*Ck*) for overexpression of *hbd* (*Ck*)
MΔ*cat*1::*adh*E2	M mutant with the *cat*1 knockout and *adh*E2 knock-in on the genome using pMTL83153-Δ*cat*1::*adh*E2
MΔ*cat*1::*adh*E2-P*cat*1-*hbd* (*Ck*)	MΔ*cat*1::*adh*E2 transformed with pMTL82151-P*cat*1-*hbd* (*Ck*) for overexpression of *hbd* (*Ck*)
MΔ*cat*1::*adh*E2-P*bgal*-*hbd* (*Ck*)	MΔ*cat*1::*adh*E2 transformed with pMTL82151-P*bgal*-*hbd* (*Ck*) for inducible expression of *hbd* (*Ck*)
plasmids	
PMTL82151	ColE1 ori, *Cm* ^R^, pBP1 ori; TraJ
PMTL83151	ColE1 ori, *Cm* ^R^, pCB102 ori; TraJ
PMTL85151	ColE1 ori, *Cm* ^R^, pIM13 ori; TraJ
PMTL83151-P*cat*1-*upp*	pMTL83151 derivative containing P*cat*1-*upp*
pMTL83151Δ*rease*	pMTL83151 derivative, targeting restriction modification system subunit
pMTL85151ΔI	pMTL85151 derivative, targeting native plasmid gene
pMTL85151Δ*upp*	pMTL85151 derivative, targeting *upp* gene
pMTL82151-Δ*cat*1::*adh*E2	pMTL82151 derivative, targeting *cat*1 for *adh*E2 insertion with the promoter P*cat*1 for *adh*E2 expression
pMTL83151-Δ*cat*1::*adh*E2	pMTL83151 derivative, targeting *cat*1 for *adh*E2 insertion with the promoter P*cat*1 for *adh*E2 expression
pMTL82151-P*cat*1-*hbd* (*Ck*)	pMTL82151 derivative, for *hbd* (*Ck*) expression with the promoter P*cat*1
pMTL82151-P*bgal*-*hbd* (*Ck*)	pMTL82151 derivative, for *hbd* (*Ck*) expression with the inducible promoter P*bgal*

To construct the mutant strains, plasmids were transformed into *C. tyrobutyricum* via conjugation.^16,69^ Briefly, the donner cells from the overnight culture of *E. coli* CA434 and *C. tyrobutyricum* recipient cells were mixed and incubated on RCM agar plates at 37 °C for 1 day. The cells were then replated on RCM agar plates containing the antibiotics for selection of transformant cells. Positive colonies were confirmed by colony PCR using primers (Table S1) in the flanking regions of homologous arms.

*C. tyrobutyricum* WT was first modified to create the M strain by knocking out the restriction modification system, native plasmid, and *upp* gene sequentially, using the CRSPR-Cas plasmids pMTL83151Δ*rease*, pMTL85151ΔI, and pMTL85151Δ*upp*, respectively. CRSPR-Cas genome-editing plasmids were cured or removed using the *upp*/5-FU counter-selection protocol.^70^ Then, the strain MΔ*cat*1::*adh*E2 was constructed by deleting *cat*1 and inserting *adh*E2 in the genome of the M strain. Finally, MΔ*cat*1::*adh*E2-P*bgal*-*hbd*(*Ck*) expressing the exogenous genes *hbd*(*Ck*), cloned from *C*. *kluyveri*, under the inducible promoter P*bgal* was constructed by transforming MΔ*cat*1:*adh*E2 with plasmid pMTL82151-P*bgal*-*hbd*(*Ck*). Removing the modification system and native plasmid improved the genetic tractability and transformation efficiency for efficient strain construction, whereas *upp* gene deletion enabled plasmid curing for subsequent genome-engineering cycles. The deletion of *cat*1 and genomic integration of *adh*E2 reduced the level of butyrate biosynthesis to almost zero and promoted stable butanol production.

### 4.3 Batch Fermentation

Unless otherwise noted, batch fermentation was carried out in 100 mL serum bottles each containing 50 mL of CGM with 60 g/L glucose and 40 g/L CaCO_3_ to evaluate the effects of *hbd*(*Ck*) expression on butanol production. After sparging with N_2_ to remove oxygen, autoclaving for sterilization, and cooling to the room temperature, each serum bottle was inoculated at 5% (v/v) with a freshly prepared seed culture and then placed in an static incubator at 37 °C. Lactose at the final concentration of 40 mM, which could induce gene expression at ∼40-fold,^30^ was added at 0, 12, or 24 h after inoculation to induce *hbd*(*Ck*) expression. Lactose as an inducer was not consumed or used by *C. tyrobutyricum* in the fermentation. All fermentation conditions were studied in duplicate serum bottles.

Batch fermentation was then studied in 2 L stirred-tank bioreactors. The bioreactor containing 850 mL of CGM was autoclaved at 121 °C and 15 psig for 30 min, mixed with 100 mL of sterilized glucose solution (600 g/L), and sparged with nitrogen gas for 30 min. Then, the reactor was inoculated with a freshly prepared seed culture (50 mL). Lactose (final concentration: 40 mM) was added at the beginning or during the early or mid-exponential phase to induce the expression of *hbd*(*Ck*) when the cell density had reached a predetermined level. The bioreactor was operated at 37 °C, 100 rpm agitation, and pH ∼ 5.5 controlled with a 30% (v/v) ammonium solution. Broth samples were taken periodically to monitor cell optical density (OD_600_) and then stored in a freezer for later analysis of glucose and metabolic products, including butanol, butyrate, ethanol, acetate, lactate, and 2,3-butanediol (2,3-BDO).

### 4.4 Effects of hbd­(Ck) Expression on Cell Growth

The effects of *hbd*(*Ck*) expression on the growth of *C. tyrobutyricum* were studied by comparing the growth kinetics of MΔ*cat*1:*adh*E2 and its derivative strains expressing *hbd*(*Ck*) under the P*cat*1 or P*bgal* control, respectively. Briefly, cells were cultured in 25 mL serum tubes each containing 15 mL of CGM with 30 g/L glucose as the substrate at 37 °C. For MΔ*cat*1::*adh*E2-P*bgal*-*hbd*(*Ck*), lactose (40 mM) was added at 0 or 12 h to induce *hbd*(*Ck*) expression. Cell growth was monitored by measuring the optical density at 600 nm (OD_600_) directly in serum tubes by using a Thermo Scientific GENESYS 40 spectrophotometer for the initial 12 h. Cell growth after 12 h was monitored by withdrawing ∼1 mL of the culture sample, which was diluted 5-fold with deionized water for OD_600_ readings. Each condition was studied in two biological replicates.

### 4.5 Intracellular NAD^+^/NADH and NADP^+^/NADPH

The intracellular NAD^+^/NADH and NADP^+^/NADPH concentrations were assayed using commercial kits #MAK468 and #MAK479, respectively, from Sigma-Aldrich. The assays were based on cycling reactions catalyzed by enzymes (lactate dehydrogenase for NAD^+^/NADH and glucose dehydrogenase for NADP^+^/NADPH). The color intensity of the reduced product of MTT via the formed NAD­(P)H in the reaction was then measured at 565 nm, which was proportional to the NAD­(P)^+^/NAD­(P)H concentration in the sample. Briefly, cells in 1 mL of cell suspension (adjusted to optical density OD_600_ = 2.0) were collected and washed once with ice-cold PBS. The pelleted cells were treated with an extraction buffer, heated at 60 °C for 5 min, mixed with assay buffer (20 μL) and neutralizing buffer (100 μL), vortexed briefly, and then centrifuged at 14,000*g* for 5 min. 40 μL of the supernatant was transferred into a 96-well plate at room temperature. After addition of 80 μL of the freshly prepared working reagent containing the reaction enzymes, substrate, and MTT, the absorbance at 565 nm (OD_565_) was monitored immediately for 15 min (for NAD^+^/NADH) or 30 min (for NADP^+^/NADPH) using a spectrophotometric plate reader. After subtracting the blank, ΔOD_565_ was used to estimate the concentration (μM) of NAD^+^/NADH or NADP^+^/NADPH present in the sample by comparing to the standard curve (ΔOD_565_ vs NAD­(P)^+^ or NAD­(P)H concentration).

### 4.6 Quantitative RT-PCR

Quantitative RT-PCR was used to assay the expression levels of *hbd*(*Ck*) in cells collected during batch cultures under various conditions. Briefly, 2 mL of culture media containing the suspended cells was collected at 36 h, and cells were harvested by centrifugation. To facilitate cell lysis, cell pellets were resuspended in Buffer Y1 (1 M sorbitol, 0.1 M EDTA, pH 7.4) supplemented with lysozyme (3–5 mg/mL) and 0.1% (v/v) β-mercaptoethanol and incubated at 35 °C with gentle shaking for 30 min. Cells were then pelleted again by centrifugation, the supernatant was removed, and the pellet was washed 1–2 times with Buffer Y1 without β-mercaptoethanol and lysozyme. Total RNA was then extracted using a FastPure cell/tissue total RNA isolation Kit (Qiagen) and reverse-transcribed into cDNA using a HiScript II first Strand cDNA Synthesis Kit (Qiagen). A quantitative RT-PCR (qRT-PCR) assay was conducted using ChamQ Blue Universal SYBR qPCR Master Mix (Vazyme) with the translational GTPase coding gene (*typ*A) as the internal reference gene.^30^ The mRNA levels of the *hbd*(*Ck*) gene relative to the internal reference gene were calculated and are reported.

### 4.74.7 Metabolic Flux Analysis

Batch fermentation data were used to estimate product yields, carbon flux distributions, and reducing equivalents (mainly NADH and reduced ferredoxin Fd_re_) consumed in the production of various metabolites based on the metabolic pathways (Figure S1) and stoichiometric equations (Table S2) given in the Appendix. Detailed methods can be found elsewhere.^25,26^

### 4.8 Analytical Methods

The broth optical density at 600 nm (OD_600_) in the bioreactor was measured after proper dilution with deionized water by using a spectrophotometer. Glucose, butyrate, acetate, and lactate were measured by high-performance liquid chromatography (HPLC), whereas butanol, ethanol, and 2,3-BDO were measured by gas chromatography (GC). Details on HPLC and GC methods can be found elsewhere.^26,71^

## Supplementary Material



## Notes

PCR primers used for plasmids construction and qPCR, stoichiometric equations for key reactions used in metabolic flux analysis (MFA), metabolic pathways in *C. tyrobutyricum* with *cat*1 knockout and *adh*E2 and *hbd*(Ck) overexpression, carbon flux distributions in the metabolic pathway for *C. tyrobutyricum* MΔcat1::*adh*E2-pbgal-*hbd*(*Ck*) in batch fermentations, effects of the lactose induction level on fermentation kinetics, and *hbd*(*Ck*) gene expression levels (PDF)

## Abbreviations

AAD: aldehyde/alcohol dehydrogenase
ABE: acetone–butanol–ethanol
CGM: clostridial growth medium
HBD: 3-hydroxybutyryl-CoA dehydrogenase
MFA: metabolic flux analysis
MV: methyl viologen
PBS: phosphate buffered saline
RCM: reinforced clostridial medium
RT-PCR: reverse-transcription polymerase chain reaction
TRY: titer, rate (productivity), yield

## References

[1] Trindade W. R. d. S., Santos R. G. d. (2017). Review on the characteristics of butanol, its production
and use as fuel in internal combustion engines. Renew. Sustain. Energy Rev..

[2] Zhao, J. ; Lu, C. ; Chen, C. C. ; Yang, S. T. Biological production of butanol and higher alcohols. In Bioprocessing Technologies in Biorefinery for Sustainable Production of Fuels, Chemicals, and Polymers; Yang, S. T. , El-Enshasy, H. A. , Thongchul, N. , Eds.; John Wiley & Sons: New York, NY, USA, 2013; pp 235–261.10.1002/9781118642047.

[3] Moon H. G., Jang Y. S., Cho C., Lee J., Binkley R., Lee S. Y. (2016). One hundred years of clostridial butanol fermentation. FEMS Microbiol. Lett..

[4] Xue C., Zhao X. Q., Liu C. G., Chen L. J., Bai F. W. (2013). Prospective
and development of butanol as an advanced biofuel. Biotechnol. Adv..

[5] Xue C., Liu M., Guo X.-W., Hudson E. P., Chen L.-J., Bai F.-W., Liu F., Yang S. T. (2017). Bridging the chemical- and bio-catalysis: high-valued
liquid transportation fuels production from renewable agricultural
residues. Green Chem..

[6] Cheng C., Bao T., Yang S. T. (2019). Engineering *Clostridium* for improved
solvent production: recent progress and perspective. Appl. Microbiol. Biotechnol..

[7] Jiménez-Bonilla P., Wang S., Whitfield T., Blersch D., Wang Y., Gonzalez-de-Bashan L.-E., Luo W., Wang Y. (2024). Tolerance
in solventogenic clostridia for enhanced butanol production: genetic
mechanisms and recent strain engineering advances. Synth. Biol. Eng..

[8] Moore C., Wang Q., Wang G., Yang S. T. (2025). Recent advances
and challenges in engineering metabolic pathways and cofactor regeneration
systems for enhanced butanol biosynthesis. Synth.
Biol. Eng..

[9] Wang J., Yang X., Chen C. C., Yang S. T. (2014). Engineering
clostridia
for butanol production from biorenewable resources: from cells to
process integration. Curr. Opin. Chem. Eng..

[10] Chang W. L., Hou W., Xu M., Yang S. T. (2022). High-rate continuous n-butanol production
by *Clostridium acetobutylicum* from glucose and butyric
acid in a single-pass fibrous-bed bioreactor. Biotechnol. Bioeng..

[11] Jiang W. Y., Zhao J. B., Wang Z. Q., Yang S. T. (2014). Stable high-titer
n-butanol production from sucrose and sugarcane juice by *Clostridium
acetobutylicum* JB200 in repeated batch fermentations. Bioresour. Technol..

[12] Diallo M., Kengen S. W. M., Lopez-Contreras A. M. (2021). Sporulation
in solventogenic and
acetogenic clostridia. Appl. Microbiol. Biotechnol..

[13] Du G., Zhu C., Xu M., Wang L., Yang S. T., Xue C. (2021). Energy-efficient
butanol production by *Clostridium acetobutylicum* with
histidine kinase knockouts to improve strain tolerance and process
robustness. Green Chem..

[14] Xu M., Zhao J., Yu L., Tang I. C., Xue C., Yang S. T. (2015). Engineering *Clostridium acetobutylicum* with a histidine kinase knockout for enhanced n-butanol tolerance
and production. Appl. Microbiol. Biotechnol..

[15] Bao T., Feng J., Jiang W., Fu H., Wang J., Yang S. T. (2020). Recent advances in n-butanol and
butyrate production
using engineered *Clostridium tyrobutyricum*. World J. Microbiol. Biotechnol..

[16] Yu M., Du Y., Jiang W., Chang W. L., Yang S. T., Tang I. C. (2012). Effects
of different replicons in conjugative plasmids on transformation efficiency,
plasmid stability, gene expression and n-butanol biosynthesis in *Clostridium tyrobutyricum*. Appl. Microbiol.
Biotechnol..

[17] Yu M., Zhang Y., Tang I. C., Yang S. T. (2011). Metabolic engineering
of *Clostridium tyrobutyricum* for n-butanol production. Metab. Eng..

[18] Du Y., Jiang W. Y., Yu M., Tang I. C., Yang S. T. (2015). Metabolic
process engineering of *Clostridium tyrobutyricum* Δack-adhE2
for enhanced n-butanol production from glucose: effects of methyl
viologen on NADH availability, flux distribution, and fermentation
kinetics. Biotechnol. Bioeng..

[19] Huang J., Du Y., Bao T., Lin M., Wang J., Yang S. T. (2019). Production
of n-butanol from cassava bagasse hydrolysate by engineered *Clostridium tyrobutyricum* overexpressing adhE2: kinetics
and cost analysis. Bioresour. Technol..

[20] Li J., Du Y., Bao T., Dong J., Lin M., Shim H., Yang S. T. (2019). n-Butanol
production from lignocellulosic biomass hydrolysates
without detoxification by *Clostridium tyrobutyricum* Δack-adhE2 in a fibrous-bed bioreactor. Bioresour. Technol..

[21] Yu L., Xu M. R., Tang I. C., Yang S. T. (2015). Metabolic engineering
of *Clostridium tyrobutyricum* for n-butanol production
through co-utilization of glucose and xylose. Biotechnol. Bioeng..

[22] Cao X., Chen Z., Liang L., Guo L., Jiang Z., Tang F., Yun Y., Wang Y. (2020). Co-valorization
of
paper mill sludge and corn steep liquor for enhanced n-butanol production
with *Clostridium tyrobutyricum* Δcat1::adhE2. Bioresour. Technol..

[23] Zhang J., Zong W., Hong W., Zhang Z. T., Wang Y. (2018). Exploiting
endogenous CRISPR-Cas system for multiplex genome editing in *Clostridium tyrobutyricum* and engineer the strain for high-level
butanol production. Metab. Eng..

[24] Nguyen N. P. T., Raynaud C., Meynial-Salles I., Soucaille P. (2018). Reviving the
Weizmann process for commercial n-butanol production. Nat. Commun..

[25] Bao T., Hou W. J., Wu X., Lu L., Zhang X., Yang S. T. (2021). Engineering *Clostridium cellulovorans* for highly selective n-butanol production from cellulose in consolidated
bioprocessing. Biotechnol. Bioeng..

[26] Feng J., Wang Q., Guo X., Hu J., Wang G., Lu L., Qin Z., Fu H., Wang J., Yang S. T. (2025). Metabolic
engineering of *Clostridium tyrobutyricum* for high-yield
n-butanol production by increasing intracellular reducing equivalent
with NADPH-dependent 3-hydroxybutyryl-CoA dehydrogenase. ACS Synth. Biol..

[27] Chen L., Xing X., Zhang P., Chen L., Pei H. (2024). Homeostatic
regulation of NAD­(H) and NADP­(H) in cells. Genes
Dis..

[28] Keasling J. D. (2010). Manufacturing
molecules through metabolic engineering. Science.

[29] Jiang T., Li C., Teng Y., Zhang J., Alexis Logan D., Yan Y. (2023). Dynamic metabolic control: from the
perspective of regulation logic. Synth. Biol.
Eng..

[30] Feng J., Wang Q., Qin Z., Guo X., Fu H., Yang S. T., Wang J. F. (2024). Development of inducible promoters
for regulating gene expression in *Clostridium tyrobutyricum* for biobutanol production. Biotechnol. Bioeng..

[31] Lee J., Jang Y. S., Han M. J., Kim J. Y., Lee S. Y. (2016). Deciphering *Clostridium tyrobutyricum* metabolism based on the whole-genome
sequence and proteome analyses. mBio.

[32] Xiao W., Wang R. S., Handy D. E., Loscalzo J. (2018). NAD­(H) and
NADP­(H)
redox couples and cellular energy metabolism. Antioxid. Redox Signaling.

[33] Liu J., Li H., Zhao G., Caiyin Q., Qiao J. (2018). Redox cofactor engineering
in industrial microorganisms: strategies, recent applications and
future directions. J. Ind. Microbiol. Biotechnol..

[34] Wang M., Chen B., Fang Y., Tan T. (2017). Cofactor engineering
for more efficient production of chemicals and biofuels. Biotechnol. Adv..

[35] Mao J., Zhang H., Chen Y., Wei L., Liu J., Nielsen J., Chen Y., Xu N. (2024). Relieving metabolic
burden to improve robustness and bioproduction by industrial microorganisms. Biotechnol. Adv..

[36] Peguin S., Goma G., Delorme P., Soucaille P. (1994). Metabolic
flexibility of *Clostridium acetobutylicum* in response
to methyl viologen addition. Appl. Microbiol.
Biotechnol..

[37] Fu H., Lin M., Tang I. C., Wang J., Yang S. T. (2021). Effects of benzyl
viologen on increasing NADH availability, acetate assimilation, and
butyric acid production by *Clostridium tyrobutyricum*. Biotechnol. Bioeng..

[38] Ma C., Kojima K., Xu N., Mobley J., Zhou L., Yang S. T., Liu X. M. (2015). Comparative proteomics analysis of
high n-butanol producing metabolically engineered *Clostridium
tyrobutyricum*. J. Biotechnol..

[39] Qi F., Thakker C., Zhu F., Pena M., San K. Y., Bennett G. N. (2018). Improvement of butanol
production in *Clostridium
acetobutylicum* through enhancement of NAD­(P)H availability. J. Ind. Microbiol. Biotechnol..

[40] Liu T., Guo R., Wang X., Gu N., Wu N., Wu J., Wang Y. (2025). Enhanced butanol production through intracellular NADH
regeneration
in CdSe-*C. acetobutylicum* biohybrid system. Bioresour. Technol..

[41] Zhao Q., Li Y., Shen B., Zhao Q., Zhu L., Jiang L. (2023). UiO-66-mediated
light-driven regeneration of intracellular NADH in *Clostridium
tyrobutyricum* to strengthen butyrate production. ACS Sustainable Chem. Eng..

[42] Ramió-Pujol S., Ganigué R., Bañeras L., Colprim J. (2015). Incubation at 25 °C
prevents acid crash and enhances alcohol production in *Clostridium
carboxidivorans* P7. Bioresour. Technol..

[43] Wang Z., Jin Y., Yang S. T. (2015). High cell
density propionic acid fermentation with
an acid tolerant strain of *Propionibacterium acidipropionici*. Biotechnol. Bioeng..

[44] Bogdahn M., Andreesen J. R., Kleiner D. (1983). Pathways and regulation of N_2_, ammonium, and glutamate assimilation by *Clostridium
formicoaceticum*. Arch. Microbiol..

[45] Kanamori K., Weiss R. L., Roberts J. D. (1989). Ammonia assimilation pathways in
nitrogen-fixing *Clostridium kluyveri* and *Clostridium butyricum*. J. Bacteriol..

[46] Wang C., Xin F. X., Kong X. P., Zhao J., Dong W. L., Zhang W. M., Ma J. F., Wu H., Jiang M. (2018). Enhanced isopropanol–butanol–ethanol
mixture production through manipulation of intracellular NAD­(P)H level
in the recombinant *Clostridium acetobutylicum* XY16. Biotechnol. Biofuels.

[47] Foulquier C., Rivière A., Heulot M., Dos Reis S., Perdu C., Girbal L., Pinault M., Dusséaux S., Yoo M., Soucaille P., Meynial-Salles I. (2022). Molecular characterization of the
missing electron pathways for butanol synthesis in *Clostridium
acetobutylicum*. Nat. Commun..

[48] Zaslaver A., Mayo A. E., Rosenberg R., Bashkin P., Sberro H., Tsalyuk M., Surette M. G., Alon U. (2004). Just-in-time transcription
program in metabolic pathways. Nat. Genet..

[49] Chubukov V., Zuleta I. A., Li H. (2012). Regulatory architecture determines
optimal regulation of gene expression in metabolic pathways. Proc. Natl. Acad. Sci. U.S.A..

[50] Iyyappan J., Bharathiraja B., Varjani S., Praveen Kumar R., Muthu Kumar S. (2022). Anaerobic biobutanol production from black strap molasses
using *Clostridium acetobutylicum* MTCC11274: media
engineering and kinetic analysis. Bioresour.
Technol..

[51] Mehrabi Z., Taheri-Kafrani A., Razmjou A., Cai D., Amiri H. (2025). Enhancing
biobutanol production by optimizing acetone-butanol-ethanol fermentation
from sorghum grains through strategic immobilization of amylolytic
enzymes. Bioresour. Technol..

[52] Zhou Z., Ding H., Shi C., Peng S., Zhu B., An X., Li H. (2025). Enhanced butanol tolerance and production from puerariae
slag hydrolysate by *Clostridium beijerinckii* through
metabolic engineering and process regulation strategies. Bioresour. Technol..

[53] Kim S., Jang Y. S., Ha S. C., Ahn J. W., Kim E. J., Hong Lim J., Cho C., Shin Ryu Y., Kuk Lee S., Lee S. Y., Kim K. J. (2015). Redox-switch
regulatory mechanism
of thiolase from *Clostridium acetobutylicum*. Nat. Commun..

[54] Tian L., Conway P. M., Cervenka N. D., Cui J., Maloney M., Olson D. G., Lynd L. R. (2019). Metabolic engineering
of *Clostridium thermocellum* for n-butanol production
from cellulose. Biotechnol. Biofuels.

[55] Shen C. R., Lan E. I., Dekishima Y., Baez A., Cho K. M., Liao J. C. (2011). Driving forces enable
high-titer anaerobic 1-butanol
synthesis in Escherichia coli. Appl. Environ.
Microbiol..

[56] Atsumi S., Cann A. F., Connor M. R., Shen C. R., Smith K. M., Brynildsen M. P., Chou K. J., Hanai T., Liao J. C. (2008). Metabolic
engineering of *Escherichia coli* for 1-butanol production. Metab. Eng..

[57] Ohtake T., Pontrelli S., Laviña W. A., Liao J. C., Putri S. P., Fukusaki E. (2017). Metabolomics-driven
approach to solving a CoA imbalance
for improved 1-butanol production in *Escherichia coli*. Metab. Eng..

[58] Bond-Watts B. B., Bellerose R. J., Chang M. C. (2011). Enzyme mechanism
as a kinetic control
element for designing synthetic biofuel pathways. Nat. Chem. Biol..

[59] Hu K., Zhao M., Zhang T. L., Zha M. W., Zhong C., Jiang Y., Ding J. P. (2013). Structures
of trans-2-enoyl reductases
from *Clostridium acetobutylicum* and *Treponema
denticola*: insights into the substrate specificity and the
catalytic mechanism. Biochem. J..

[60] Vemuri G. N., Altman E., Sangurdekar D. P., Khodursky A. B., Eiteman M. A. (2006). Overflow metabolism in *Escherichia coli* during steady-state growth: transcriptional regulation and effect
of the redox ratio. Appl. Environ. Microbiol..

[61] San K., Bennett G., Berríos-Rivera S. J., Vadali R. V., Yang Y. T., Horton E., Rudolph F. B., Sariyar B., Blackwood K., Blackwood K. (2002). Metabolic
engineering through cofactor manipulation and its effects on metabolic
flux redistribution in *Escherichia coli*. Metab. Eng..

[62] Lopez
de Felipe F., Kleerebezem M., de Vos W. M., Hugenholtz J. (1998). Cofactor engineering:
a novel approach to metabolic engineering in *Lactococcus lactis* by controlled expression of NADH oxidase. J. Bacteriol..

[63] Li S., Ye Z., Moreb E. A., Hennigan J. N., Castellanos D. B., Yang T., Lynch M. D. (2021). Dynamic
control over feedback regulatory
mechanisms improves NADPH flux and xylitol biosynthesis in engineered *E. coli*. Metab. Eng..

[64] Jiang T., Li C., Teng Y., Zhang J., Alexis Logan D., Yan Y. (2023). Dynamic metabolic control: From the
perspective of regulation logic. Synth. Biol.
Eng..

[65] Moore C., Wang Q., Wang G., Feng J., Qin Z., Yang S. T. (2025). In-silico analysis and engineering of an aldehyde/alcohol
dehydrogenase for alternative cofactor utilization and selective butanol
production. ACS Synth. Biol..

[66] Zou S. P., Ding W., Han Y. Y., Niu K., Xue Y. P., Zheng Y. G. (2024). Efficient L-phosphinothricin production
by engineered *Escherichia coli* co-expressing glutamate
dehydrogenase,
glucose dehydrogenase and NAD kinase with NADPH regeneration. Biochem. Eng. J..

[67] Hu L., Huang H., Yuan H., Tao F., Xie H., Wang S. (2016). Rex in *Clostridium kluyveri* is a global redox-sensing
transcriptional regulator. J. Biotechnol..

[68] Heap J. T., Pennington O. J., Cartman S. T., Minton N. P. (2009). A modular system
for *Clostridium* shuttle plasmids. J. Microbiol. Methods.

[69] Fu H., Yu L., Lin M., Wang J., Xiu Z., Yang S. T. (2017). Metabolic
engineering of *Clostridium tyrobutyricum* for enhanced
butyric acid production from glucose and xylose. Metab. Eng..

[70] Guo X., Zhang H., Feng J., Yang L., Luo K., Fu H., Wang J. (2023). De novo biosynthesis
of butyl butyrate in engineered *Clostridium tyrobutyricum*. Metab.
Eng..

[71] Qin Z., Feng J., Li Y., Zheng Y., Moore C., Yang S. T. (2024). Engineering the
reductive tricarboxylic acid pathway
in *Aureobasidium pullulans* for enhanced biosynthesis
of poly-L-malic acid. Bioresour. Technol..

