# Supplementary material for: Engineering Clostridium Tyrobutyricum for High Butanol Production through Induction Expression of Exogenous NADPH-Dependent HBD
Source: ACS Synth Biol. 2026 Mar 11;15(3):1116–29. doi: 10.1021/acssynbio.5c00792 (PMC13010368; doi:10.1021/acssynbio.5c00792)
Supplement: Supplementary file 1 [file sb5c00792_si_001.pdf]

Supplementary Information

Engineering *Clostridium tyrobutyricum* for high butanol production through induction expression of exogenous NADPH-dependent HBD

Qingke Wang[a], Geng Wang[a], Jialei Hu,[a] Jun Feng[a], Ziqi Emily Lin[a], Shang-Tian Yang[a,*]

[a] William G. Lowrie Department of Chemical and Biomolecular Engineering, The Ohio State University, Columbus, Ohio 43210, USA

Funding:

The Advanced Research Projects Agency–Energy (ARPA-E) (Grant number: DE-AR0001512)

The Department of Energy EERE – BETO (Grant number: DE-EE0010300)

The US Department of Agriculture - National Institute of Food & Agriculture (NIFA) (Award Number: 2022-67021-37606)

The National Science Foundation (Award Number 2328215).

*Corresponding author:

Shang-Tian Yang, Department of Chemical and Biomolecular Engineering, The Ohio State University, 151 West Woodruff Avenue, Columbus, Ohio 43210, USA; Phone: 1-614-292-6611; E-mail: yang.15@osu.edu

Additional authors:

Qingke Wang, E-mail: qingke16543@gmail.com

Geng Wang, E-mail: geng97774@gmail.com

Jialei Hu, E-mail: hu.2536@osu.edu

Jun Feng, E-mail: fengjun1399@126.com

Ziqi Emily Lin, E-mail: lin.2155@buckeyemail.osu.edu

Submitted to *ACS Synthetic Biology*

Table S1. PCR primers used in this study

Primers	Sequences (5′-3′)
Plac-F	GAAACAGCTATGACCGCGGCCGCTTATATACTTGGTTTATTTACTTGATTATT
spacer-renase-R	CGATGTATCAGGATATACACAGGATGTTACTAGTCGTTATTTAAATACATCTCATGTTAAGGT
spacer-renase-F	AACGACTAGTAACATCCTGTGTATATCCTGATACATCGGTTGAACCTTAACATGAGATGT
arm-top-renase-F	TAAGCTTGGCGTAATCATGGTTCATTTCTCACTTGCCTCCTCT
arm-up-renase-R	CTTGGATATTTTATGAATTCTTATGCTGTGGCATTGAACGCTTAGTGA
Arm-down-renase-F	GCATAAGAATTCATAAAATATCCAAGG
arm-down-renase-R	CATTAGCTAAGGATTCAGAACGTGAACTGCCGACTATCAATTGAC
NM-spacer-R	TGATACAGTAATCCTATCATACGATCAATTATTTAAATACATCTCATGTTAAGGT
NM-SPACER-F	GATCGTATGATAGGATTACTGTATCAATATGTTGAACCTTAACATGAGATGTATT
NM-83-CAT1-R	TTCCATCCTTAAAGTCTACGACGTCACCATGATTACGCCAAGCTTA
new-m13-F	ATCAGGAAACAGCTATGACCGC
new-M13-R	GCGGTCATAGCTGTTTCCTGAT
cat1-ha2-R	CTCTCAGGATTGGATGTAATAG
pcat1-83-F	GACGTCGTAGACTTTAAGGATGGAA
PbgalLA-F	GACTATTCCTCCTTTCTGC
hbdCK-F2	ATTTATATGAGGAGGAATTTCAATTAAAAGTGTAGCGGTTTTAGGTA
hbdCK-R1	GCTAGCGCCATTCGCCATTCTTAATAAGCGAAGAATCCTTTTCCTGA
p82151-Pcat1-F	AAAAACCACCCTTTCATAAATTATA
P82-adhE2-s-F	GAATGGCGAATGGCGCTAGCATA
hbdCK-ter-R1	CCGGGGATCCTCTAGAGTCGATTAATAAGCGAAGAATCCTTTTCCTGA
Hbd-pcat1-F	TATAATTTATGAAAGGGTGGTTTTTATTAAAAGTGTAGCGGTTTTAGGTA
TypA-F	CAATAATTGCCCATGTAGATCACGG
TypA-R	GAGTCCATAACCCTCTCTTGAAC

Table S2. Stoichiometric equations for key reactions used in metabolic flux analysis (MFA)

Number	Equation
R1	Glucose → 2 Pyruvate + 2 NADH
R2	Pyruvate + NADH → 2,3 butanediol
R3	Pyruvate + Fd_{ox}→ Acetyl-CoA + Fd_{re} + CO_2
R4	Acetyl-CoA → Acetic acid
R5	Acetyl-CoA + 2 NADH → Ethanol
R6	2 Acetyl-CoA → Acetoacetyl-CoA
R7	Acetoacetyl-CoA + NADH → 3-hydroxybutyryl-CoA
R8	3-hydroxybutyryl-CoA + 2 NAD(P)H + Fd_{ox} → Butyryl-CoA + Fd_{re}
R9	Butyryl-CoA → Butyric acid
R10	Butyryl-CoA +2 NADH → Butanol
R11	FdH_2 ↔ NADH
R12	FdH_2 → H_2

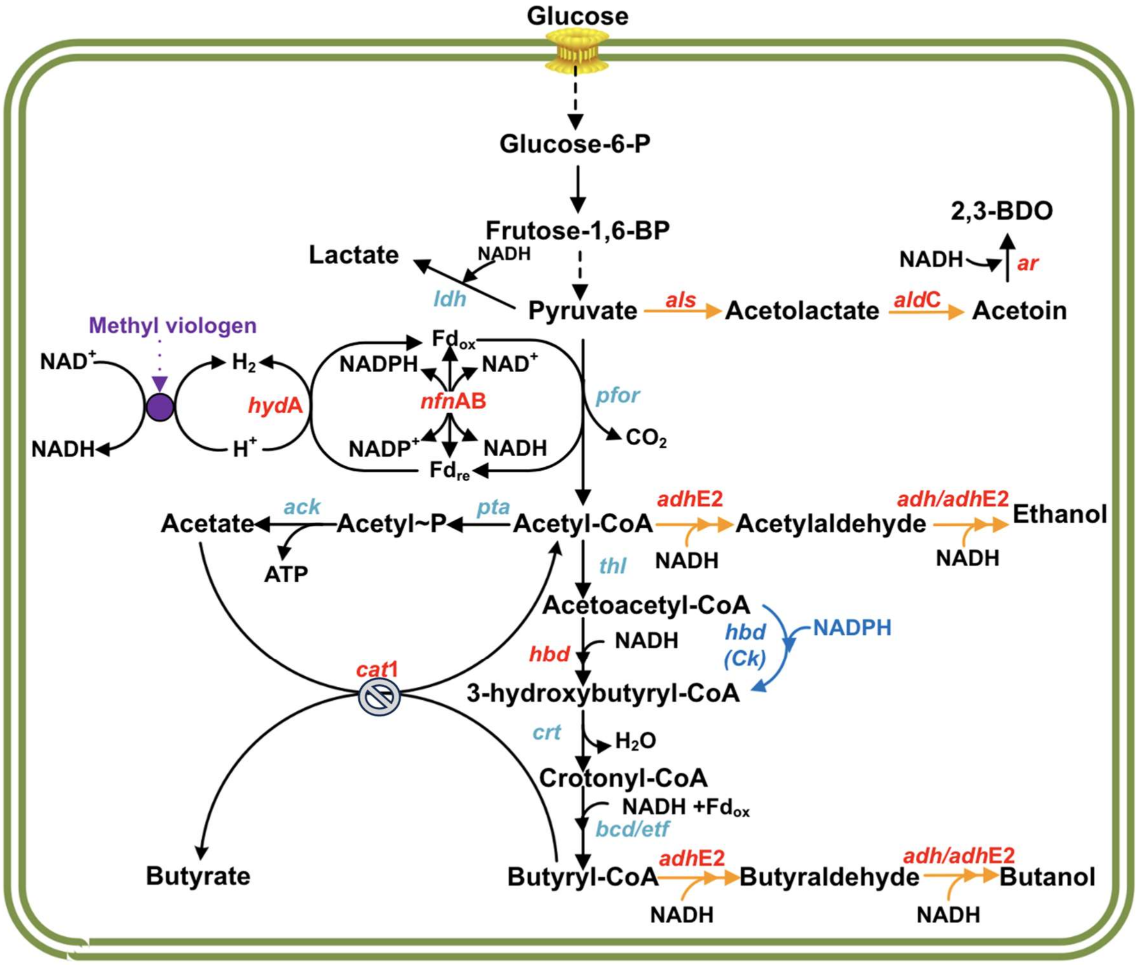

Figure S1. Metabolic pathways in *C. tyrobutyricum* MΔ*cat*1::*adh*E2-P*bgal*-*hbd*(*Ck*) with *cat*1 knockout, which disables butyrate biosynthesis, and overexpression of heterologous *adh*E2 for butanol and ethanol biosynthesis and *hbd*(*Ck*) for increased carbon flux from acetyl-CoA to butyryl-CoA. Native genes in the pathways: *ldh*: lactate dehydrogenase; *als*: acetolactate synthase; *ald*C: acetolactate decarboxylase; *ar*: acetoin reductase; *pfo*R: pyruvate:ferredoxin oxidoreductase; *hyd*A: hydrogenase; *ack*: acetate kinase; *pta*: phosphotransacetylase; *bcd*: butyryl-CoA dehydrogenase; *cat*1: butyryl-CoA/acetate CoA transferase; *thl*: thiolase; *hbd*: NADH-dependent 3-hydroxybutyryl-CoA dehydrogenase; *crt*: crotonase; *bcd*: butyryl-CoA dehydrogenase; *etf*: electron transferring flavoprotein; exogenous genes: *adh*E2: aldehyde/alcohol dehydrogenase from *C. acetobutylicum*; *hbd*(*Ck*): NADPH-dependent 3-hydroxybutyryl-CoA dehydrogenase from *C. kluyveri*.

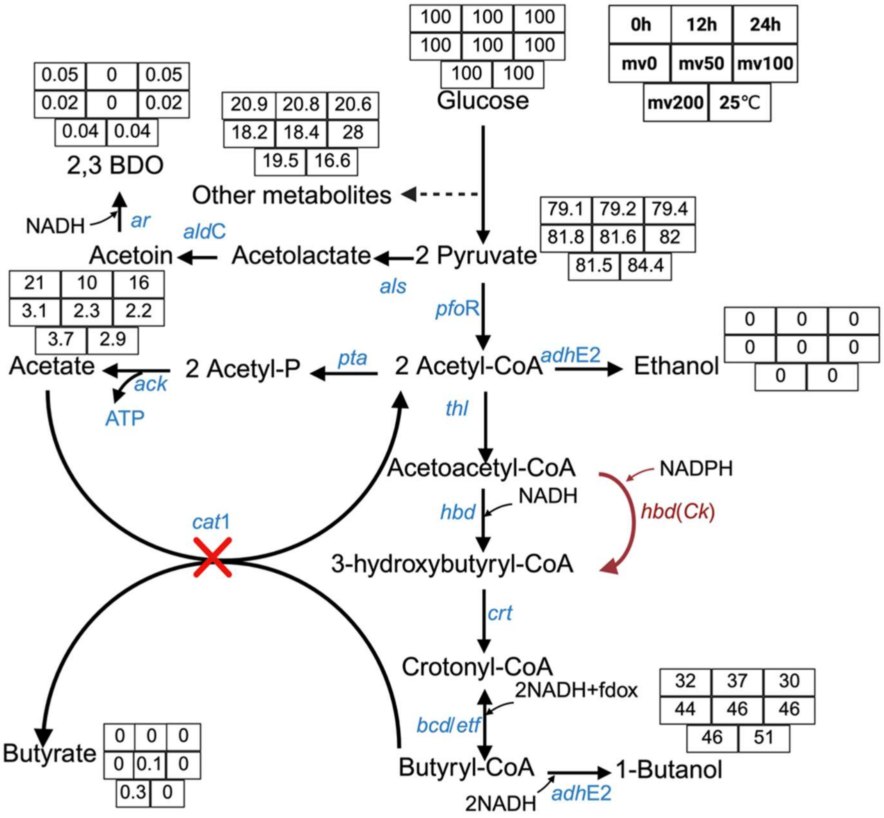

Figure S2. Carbon flux distributions in the metabolic pathway for *C. tyrobutyricum* MΔ*cat*1::*adh*E2-P*bgal*-*hbd(Ck)* in batch fermentations in serum bottles under different lactose induction times (0, 12, 24 h), MV concentrations (0, 50, 100, 200 mM) and at low temperature (25 ℃). The flux values (%) given in the boxes next to the reactions are normalized with glucose (6 carbon) consumed in the fermentation being 100%. Genes in the pathways: *als*: acetolactate synthase; *ald*C: acetolactate decarboxylase; *ar*: acetoin reductase; *ack*: acetate kinase; *pta*: phosphotransacetylase; *adh*E2: aldehyde/alcohol dehydrogenase; *pfo*R: pyruvate:ferredoxin oxidoreductase; *bcd*: butyryl-CoA dehydrogenase; *cat*1: butyryl-CoA/acetate CoA transferase; *thl*: thiolase; *hbd*: 3-hydroxybutyryl-CoA dehydrogenase; *crt*: crotonase; *bcd*: butyryl-CoA dehydrogenase; *etf*: electron transferring flavoprotein**.**

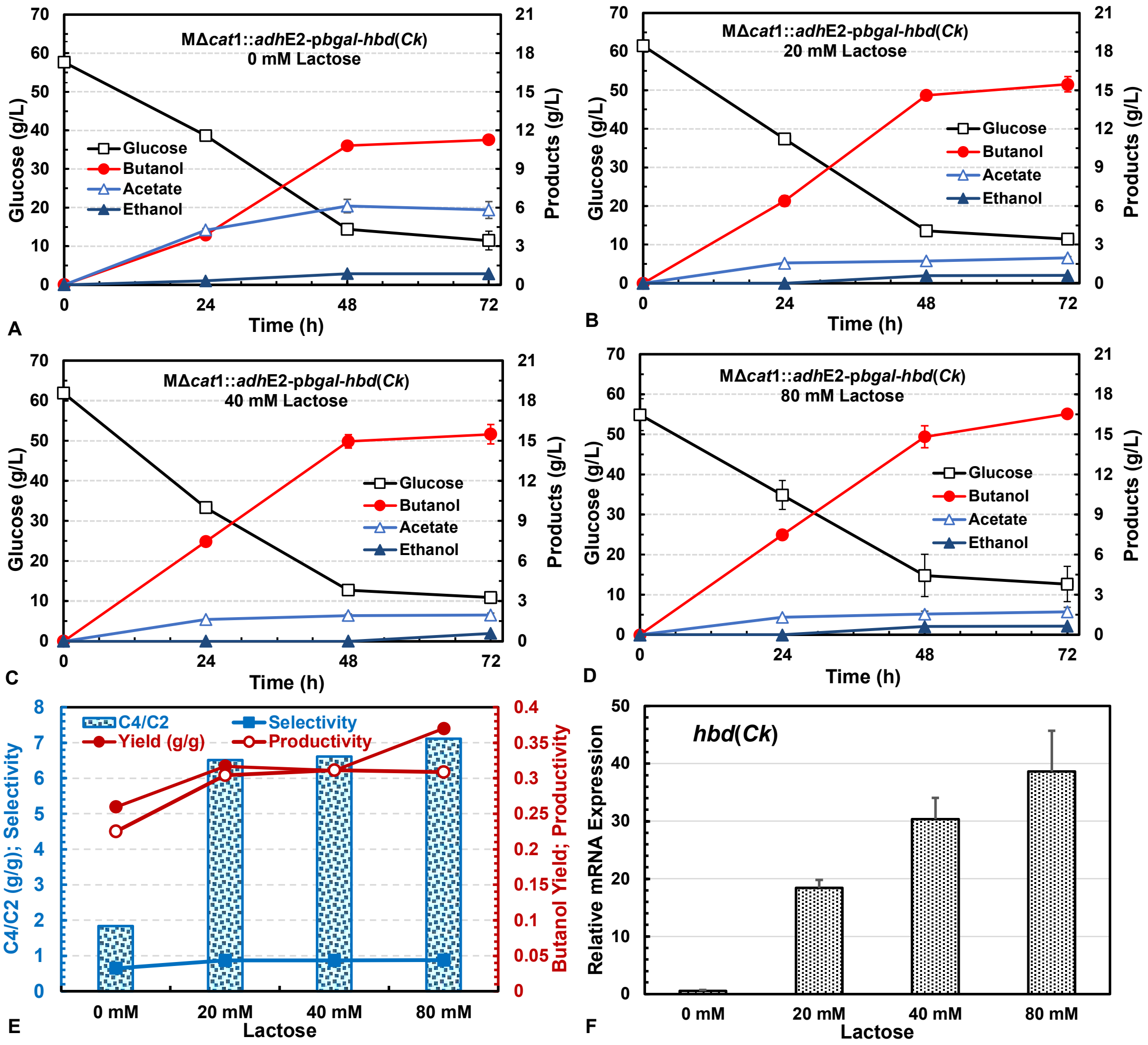

Figure S3. Effects of lactose induction level on fermentation kinetics and *hbd*(Ck) gene expression levels in MΔ*cat*1::*adh*E2-P*bgal*-*hbd*(*Ck*). (A-D) Batch fermentations with lactose induction at 12 h at zero (no lactose), 20, 40, and 80 mM, respectively. (E) Effects of lactose concentration to induce *hbd*(*Ck*) on butanol production yield, productivity, selectivity and C4/C2 ratio. (F) Relative *hbd*(*Ck*) mRNA levels to internal reference gene (*typ*A) in cells collected at 36 h of the batch fermentation with different lactose induction levels. RT-qPCR analysis showed a clear dose-dependent increase in *hbd*(*Ck*) mRNA levels with lactose (Fig. S3F), confirming that the P*bgal* system is tunable by lactose dosage. However, the seemingly higher butanol yield and C4/C2 ratio might be attributed to the grossly lower initial glucose in the fermentation medium, which had a large error in HPLC assay. Also, the higher butanol yield could not be due to the higher lactose concentration added for *hbd*(*Ck*) induction since *C. tyrobutyricum* cannot use lactose as the carbon source for growth.
